# WNT signalling control by KDM5C during development affects cognition

**DOI:** 10.1038/s41586-024-07067-y

**Published:** 2024-02-21

**Authors:** Violetta Karwacki-Neisius, Ahram Jang, Engin Cukuroglu, Albert Tai, Alan Jiao, Danilo Predes, Joon Yoon, Emily Brookes, Jiekai Chen, Aimee Iberg, Florian Halbritter, Katrin Õunap, Jozef Gecz, Thorsten M. Schlaeger, Shannan Ho Sui, Jonathan Göke, Xi He, Maria K. Lehtinen, Scott L. Pomeroy, Yang Shi

**Affiliations:** 1 Division of Newborn Medicine and Epigenetics Program, Department of Pediatrics, Boston Children’s Hospital, Harvard Medical School, Boston, MA, USA.; 2 Department of Pathology, Boston Children’s Hospital, Boston, MA, USA.; 3 Computational and Systems Biology, Genome Institute of Singapore, Singapore, Singapore.; 4 Department of Immunology, Tufts University School of Medicine, Boston, MA, USA.; 5 Data Intensive Studies Center, Tufts University, Medford, MA, USA.; 6 Ludwig Institute for Cancer Research, Nuffield Department of Medicine, University of Oxford, Oxford, UK.; 7 Department of Neurology, F. M Kirby Neurobiology Center, Boston Children’s Hospital, Harvard Medical School, Boston, MA, USA.; 8 Department of Biostatistics, The Harvard Chan School of Public Health, Bioinformatics Core, Cambridge, MA, USA.; 9 Children’s Cancer Research Institute, St Anna Kinderkrebsforschung, Vienna, Austria.; 10 Department of Clinical Genetics, Genetic and Personalized Medicine Clinic, Tartu University Hospital, Tartu, Estonia.; 11 Department of Clinical Genetics, Institute of Clinical Medicine, University of Tartu, Tartu, Estonia.; 12 Adelaide Medical School and Robinson Research Institute, University of Adelaide, Adelaide, South Australia, Australia.; 13 Stem Cell Program, Boston Children’s Hospital, Boston, MA, USA.; 14 Division of Hematology/Oncology, Boston Children’s Hospital and Dana-Farber Cancer Institute, Boston, MA, USA.; 15 Broad Institute of MIT and Harvard, Cambridge, MA, USA.; 16 Department of Neurology, Boston Children’s Hospital, Boston, MA, USA.; 17 Harvard Medical School, Boston, MA, USA.; 18 Present address: School of Biological Sciences, University of Southampton, Southampton, UK.; 19 Present address: CAS Key Laboratory of Regenerative Biology and Guangdong Provincial Key Laboratory of Stem Cell and Regenerative Medicine, Guangzhou Institutes of Biomedicine and Health, Chinese Academy of Sciences, Guangzhou, China.; 20 These authors contributed equally: Engin Cukuroglu, Albert Tai, Alan Jiao, Danilo Predes, Joon Yoon, Emily Brookes.

## Abstract

Although *KDM5C* is one of the most frequently mutated genes in X-linked intellectual disability^1^, the exact mechanisms that lead to cognitive impairment remain unknown. Here we use human patient-derived induced pluripotent stem cells and *Kdm5c* knockout mice to conduct cellular, transcriptomic, chromatin and behavioural studies. KDM5C is identified as a safeguard to ensure that neurodevelopment occurs at an appropriate timescale, the disruption of which leads to intellectual disability. Specifically, there is a developmental window during which KDM5C directly controls WNT output to regulate the timely transition of primary to intermediate progenitor cells and consequently neurogenesis. Treatment with WNT signalling modulators at specific times reveal that only a transient alteration of the canonical WNT signalling pathway is sufficient to rescue the transcriptomic and chromatin landscapes in patient-derived cells and to induce these changes in wild-type cells. Notably, WNT inhibition during this developmental period also rescues behavioural changes of *Kdm5c* knockout mice. Conversely, a single injection of WNT3A into the brains of wild-type embryonic mice cause anxiety and memory alterations. Our work identifies KDM5C as a crucial sentinel for neurodevelopment and sheds new light on *KDM5C* mutation-associated intellectual disability. The results also increase our general understanding of memory and anxiety formation, with the identification of WNT functioning in a transient nature to affect long-lasting cognitive function.

Neurodevelopment is a highly orchestrated process whereby progenitors and neurons emerge during development in a rigorously coordinated temporal and spatial order. Substantial efforts have been made to understand the role of transcriptional regulators in controlling the differentiation of neuronal cell types during early neuronal development^2-4^. However, the regulatory mechanisms that mediate the timing of the numerous molecular and cellular events vital for neuronal development and their contributions to disease development remain incompletely understood^5^.

A plethora of neurodevelopmental disorders, including intellectual disability (ID), autism spectrum disorder, attention-deficit hyperactivity disorder and cerebral palsy, which affect one in six children in the United States alone^6^, have been genetically linked to chromatin-modifying enzymes and other epigenetic regulators^7,8^. The histone H3 lysine 4 dimethyl and trimethyl specific demethylase KDM5C^9,10^ is one such crucial epigenetic regulator. Mutations in *KDM5C* are involved in X-linked ID and in autism spectrum disorder^1,11^. Thus, KDM5C provides an important paradigm for studying the neurodevelopmental causes of cognitive dysfunction.

Patients with *KDM5C* mutations suffer from mild to severe ID that is often accompanied by microcephaly, behavioural disturbance and epilepsy^12^. The *Kdm5c* knockout (KO) mouse model recapitulates many clinical features observed in human patients with *KDM5C* mutations, including learning and memory deficits^13,14^. However, how KDM5C directs neuronal differentiation and which developmental time points are affected by KDM5C loss and lead to ID phenotypes are unknown. Therefore, insights into the function of KDM5C in brain development, especially in humans, are needed and crucial for understanding the pathology of neuronal diseases. Our findings not only provide significant new insights into the fundamental mechanisms that regulate neuronal development but also decipher how events happening early in the embryo can form the basis for impairments in memory and anxiety later in life. In addition, our data highlight the canonical WNT signalling pathway as a potential therapeutic target for ID, with an unexpected role of WNT in cognition during our identified developmental window.

## Improper differentiation timing of *KDM5C* mutants

**Extended Data Fig. 1 ∣:**
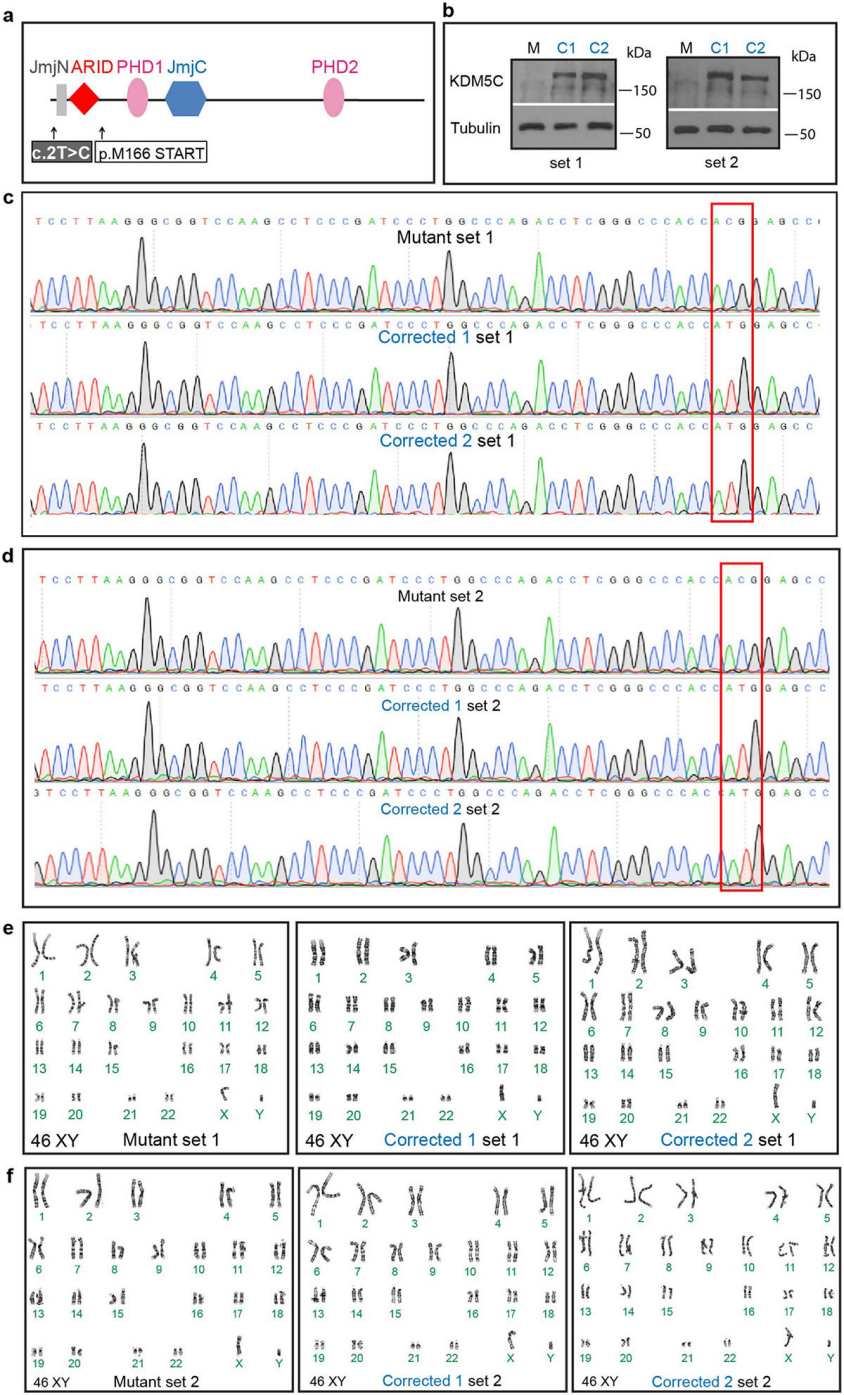
Isogenic correction of the c.2T>C in patient iPSC lines. **a**, Schematic representation of the KDM5C protein structure. Domains and the location of patient c.2T>C mutation are indicated. M166 is the predicted, alternative translational start codon for patients with the c.2T>C mutation. **b**, Western Blot analysis for KDM5C protein in patient Mutant (M) and Corrected (C1 and C2) cells in brother 1 (left, set 1) and brother 2 (right, set 2). 2 independent experiments with similar results were performed. Gels were run separately to see the entire lane stained with the KDM5C antibody in order to determine if there are any non-specific cross-reactivities of the KDM5C antibodies by comparing the Corrected cells with Mutant cells. For gel source data see Supplementary Fig. 1. **c**, Sanger sequencing results showing correction (red box) of the Mutation sequence (ACG) to the WT sequence (ATG) in Corrected 1 and Corrected 2 lines of brother 1 (set 1) and (**d**) brother 2 (set 2). **e**, Karyotype analysis of Mutant and Corrected 1 and Corrected 2 cells of brother 1 (set 1) and (**f**) brother 2 (set 2).

To study KDM5C in human neurodevelopment and disease, we obtained fibroblast cells from two brothers harbouring the c.2T>C mutation in *KDM5C* and have severe ID and developmental delay, short stature and microcephaly^15,16^ (Extended Data Fig. 1a-f). We refer to the patient-derived and the isogenic, corrected induced pluripotent stem (iPS) cell lines as ‘mutant’ and ‘corrected’, respectively.

**Extended Data Fig. 2 ∣:**
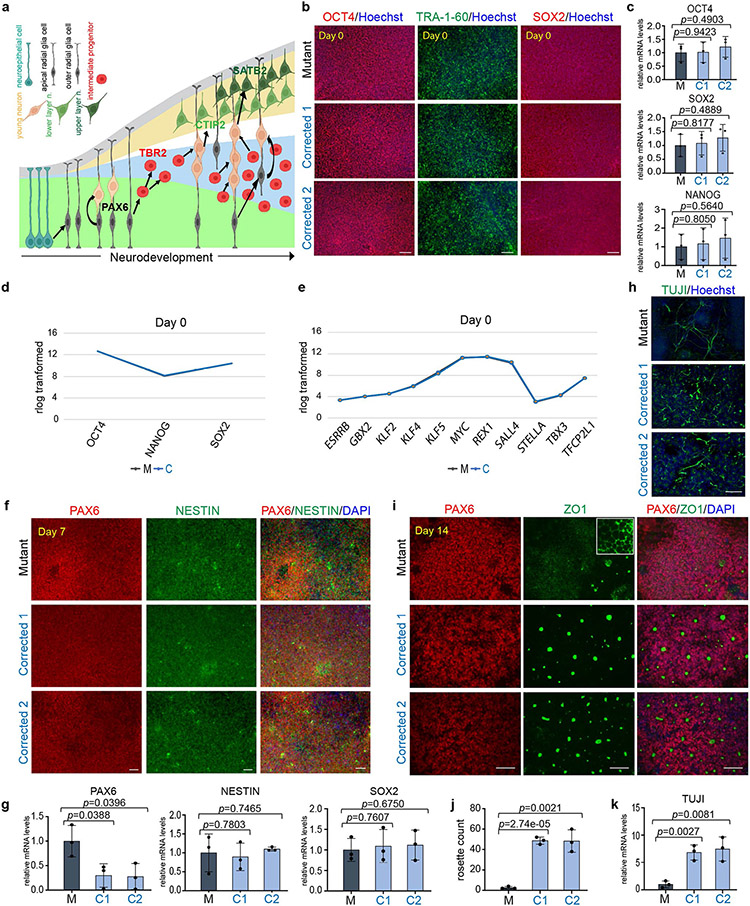
*KDM5C* mutation leads to a delay in neuronal differentiation. **a**, Simplified schematics depicting the temporal and spatial appearance of main neuronal cell types during early cortex development. Arrows indicate lineage relationships. Neuroepithelial cells give rise to apical radial glia cells (Pax6^+^) that initially divide symmetrically to generate daughter cells. During the neurogenic phase however, most apical radial glia cells divide asymmetrically and give rise to a neuron or to intermediate progenitor cells. Intermediate progenitor cells divide symmetrically in the subventricular zone to generate two daughter cells that migrate towards the CP to generate neurons. Depending on the stage of development intermediate progenitor cells can give rise to either lower layer (CTIP2^+^) or upper layer (SATB2^+^) neurons^28^. Graph was adapted from^67^ and created with BioRender. **b**, Immunofluorescence analysis for OCT4, TRA-1-60 and SOX2 in Mutant and Corrected lines at day 0 of neuronal differentiation. Cells were counterstained with Hoechst. More than 3 independent experiments were performed with similar results. **c**, qPCR analysis of *OCT4, SOX2* and *NANOG* mRNAs in Mutant (M) and Corrected lines (C1 and C2). Data are represented as mean ± SD of 3 independent experiments. The *p*-values by two-tailed unpaired Student’s t-test are indicated. *P* < 0.05 was considered statistically significant. **d**, Rlog transformed expression intensities for *OCT4, NANOG* and *SOX2* mRNAs and (**e**) eleven further pluripotency-related genes in the Mutant (M) and Corrected (C) line, extracted from RNA-seq data. **f**, Immunofluorescence for PAX6 and NESTIN at day 7 of neuronal differentiation in Mutant and two Corrected lines. Cells were counterstained with Dapi. More than 3 independent experiments were performed with similar results. **g**, qPCR analysis for *PAX6, NESTIN* and *SOX2* mRNAs at day 7. Elevation of PAX6 levels together with the phenotype described in (**h**) were the first phenotypic difference observed in our data set. Data are represented as mean ± SD of 3 independent experiments. The *p*-values by two-tailed unpaired Student’s t-test are indicated. *P* < 0.05 was considered statistically significant. **h**, Immunofluorescence for TUJI at day 11 of neuronal differentiation in Mutant and Corrected lines. Cells were counterstained with Hoechst. At day 11, a transient increase in neuronal processes was observed in the early neurons that appeared in Mutant cells. This was a short and transient phenotype that lasted for about 3 days and that affected a small number of cells. More than 3 independent experiments were performed with similar results. This data is related to Extended Data Fig. 3i that shows that this phenotype is dependent on Wnt/β-catenin signaling. **i**, Immunofluorescence for PAX6 and ZO1 at day 14 of neuronal differentiation in Mutant and two Corrected lines. Cells were counterstained with Dapi. More than 3 independent experiments were performed with similar results. **j**, rosette count in Mutant (M) and Corrected lines (C1 and C2). Data are represented as mean ± SD of 3 independent experiments. The *p*-values by two-tailed unpaired Student’s t-test are indicated. *P* < 0.05 was considered statistically significant. **k**, qPCR analysis of *TUJI* mRNAs in Mutant (M) and Corrected lines (C1 and C2). Data are represented as mean ± SD of 3 independent experiments. The *p*-values by two-tailed unpaired Student’s t-test are indicated. *P* < 0.05 was considered statistically significant. Scale bars, 100 μm.

**Extended Data Fig. 3 ∣:**
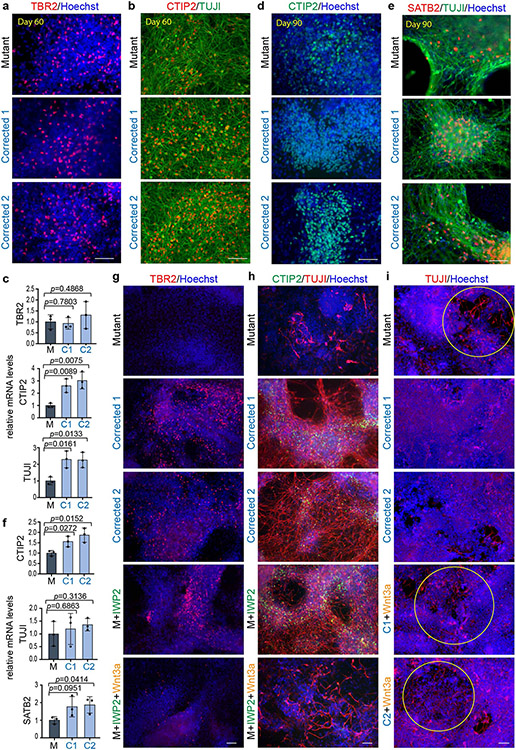
*KDM5C* patient mutation leads to a delay in neuronal differentiation and delayed differentiation can be rescued by transient downregulation of WNT/β-catenin signaling in patient Mutant cells. **a**, Immunofluorescence for TBR2 and (**b**) CTIP2 and TUJI at day 60 of neuronal differentiation in patient Mutant and Corrected lines. Cells were counterstained with Hoechst. More than 3 independent experiments were performed with similar results. Scale bars, 100 μm. **c**, qPCR analysis of *TBR2, CTIP2* and *TUJI* mRNAs in Mutant (M) and Corrected lines (C1 and C2) at day 60. Data are represented as mean ± SD of 3 independent experiments. The *p*-values by two-tailed unpaired Student’s t-test are indicated. *P* < 0.05 was considered statistically significant. **d**, Immunofluorescence for CTIP2 and (**e**) SATB2 and TUJI at day 90 of neuronal differentiation in Mutant and Corrected lines. Cells were counterstained with Hoechst. More than 3 independent experiments were performed with similar results. Scale bars, 100 μm. **f**, qPCR analysis of *CTIP2, TUJI* and *SATB2* mRNAs in Mutant (M) and Corrected lines (C1 and C2) at day 90. Data are represented as mean ± SD of 3 independent experiments. The *p*-values by two-tailed unpaired Student’s t-test are indicated. *P* < 0.05 was considered statistically significant. **g**, Immunofluorescence analysis for TBR2 and (**h**) CTIP2 and TUJI in patient Mutant and Corrected lines at day 30 of neuronal differentiation, which have been treated transiently either with vehicle (Mutant (M), Corrected 1 and Corrected 2), IWP2 (1 mM (first pulse)/0.25 mM (second pulse)) or a combination of IWP2 (1 mM) and Wnt3a (200 ng/ml). More than 3 independent experiments were performed with similar results. **i**, Immunofluorescence for TUJI at day 11 of neuronal differentiation in Mutant and Corrected lines. Cells were counterstained with Hoechst. Corrected cells cultured with Wnt3a (200 ng/ml) show regions of greater axonal outgrowth similarly as observed in Mutant cells at this time of differentiation. More than 3 independent experiments were performed with similar results. This data is related to Extended Data Fig. 2h. Abbreviations: C1=Corrected 1, C2=Corrected.

**Fig. 1 ∣:**
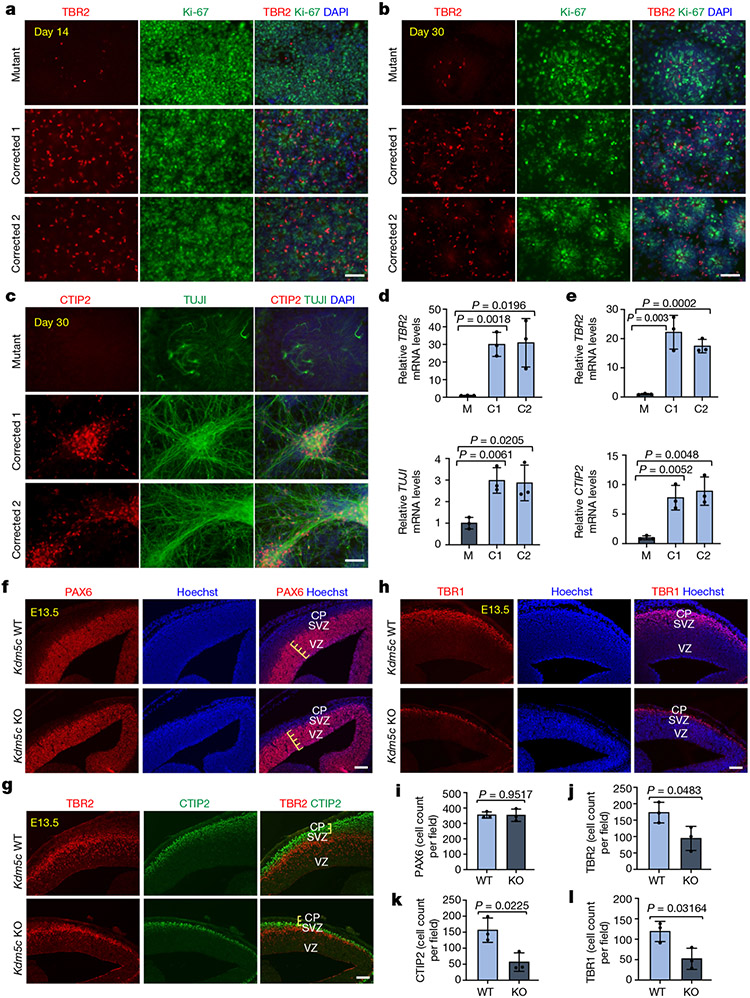
*KDM5C* mutations lead to inefficient entry into the intermediate progenitor state and delayed neuron generation. **a–c**, Immunofluorescence images of TBR2 and Ki-67 at day 14 (**a**) and day 30 (**b**) and of CTIP2 and TUJI at day 30 (**c**) of neuronal differentiation in mutant (M) and two corrected cell lines (C1 and C2). More than three independent experiments were performed with similar results. **d,e**, qPCR analysis of *TBR2* and *TUJI* at day 14 (**d**) and *TBR2* and *CTIP2* at day 30 (**e**). Data represent the mean ± s.d. of three independent experiments. **f–h**, Immunofluorescence images of PAX6 (**f**), TBR2 and CTIP2 (**g**) and TBR1 (**h**) in WT (*Kdm5c* WT) and *Kdm5c* KO mouse cortices at E13.5 of development. Cells were counterstained with Hoechst. Three independent experiments were performed with similar results. CP, cortical plate; SVZ, subventricular zone; VZ, ventricular zone. **i–l**, Positive cell count for PAX6 (**i**), TBR2 (**j**), CTIP2 (**k**) and TBR1 (**l**) in *Kdm5c* WT and *Kdm5c* KO mouse cortices. Three independent fields were assessed and reproduced in three *Kdm5c* WT and three *Kdm5c* KO animals. Data represent the mean ± s.d. For **d,e,i–l**, *P* values were calculated using two-tailed unpaired Student’s *t*-test; *P* < 0.05 was considered significant. Scale bars, 100 μm (**a–c**, **f–h**).

To define the temporal dynamics of cellular composition, we monitored the sequential appearance of major cell populations (Extended Data Fig. 2a). No significant differences were observed at the iPS cell stage between mutant and corrected cell lines with respect to the expression of pluripotency factors (Extended Data Fig. 2b-e) and primary progenitor genes (Extended Data Fig. 2f,g). Phenotypic distinctions emerged around days 7–11 (Extended Data Figs. 2f-h and 3i), with major phenotypic differences apparent from day 14 onwards. Rosette counting revealed a substantial difference in their ability to form the rosette structure (Extended Data Fig. 2i,j). Notably, the emergence of intermediate progenitors (TBR2^+^) at day 14 was readily detectable in the corrected cells but not in the mutant cells (Fig. 1a,d, top). Even at day 30 of differentiation, intermediate progenitor cells were barely detectable in the mutant cells (Fig. 1b,e, top), similar to the day 14 population (Fig. 1a,d). These results indicated that there is significant impairment in the generation of intermediate progenitors in mutant cells.

**Extended Data Fig. 4 ∣:**
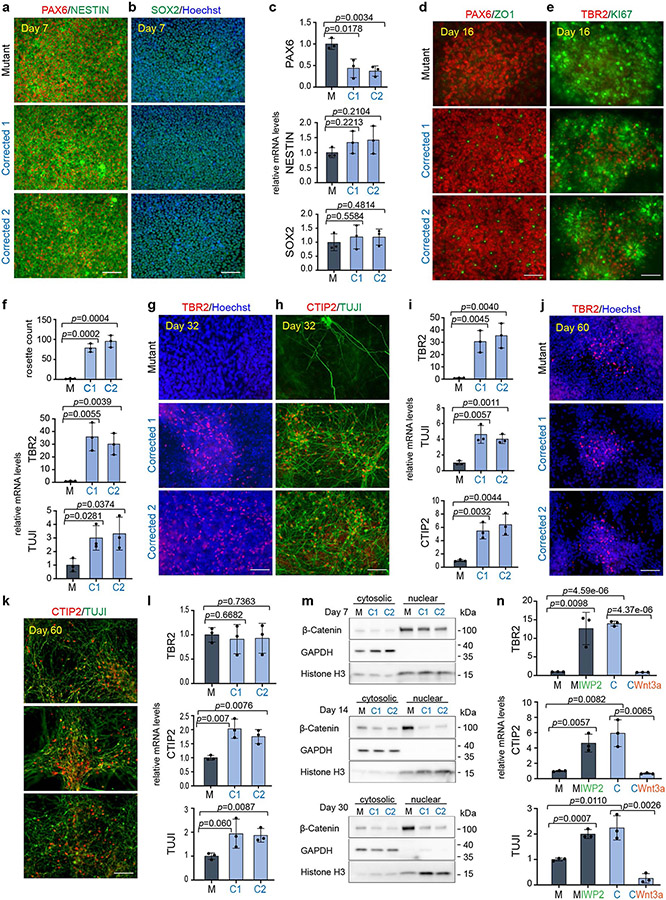
*KDM5C* mutation leads to a delay in neuronal differentiation and can be rescued and induced by canonical Wnt signaling manipulation in the second brother. **a**, Immunofluorescence for PAX6, NESTIN and (**b**) SOX2 at day 7 of neuronal differentiation in Mutant and two Corrected lines of brother 2. Cells were counterstained with Hoechst. 3 independent experiments were performed with similar results. **c**, qPCR analysis for *PAX6, NESTIN* and *SOX2* mRNAs at day 7 in the second brother. Data are represented as mean ± SD of 3 independent experiments. The *p*-values by two-tailed unpaired Student’s t-test are indicated. *P* < 0.05 was considered statistically significant. **d**, Immunofluorescence for PAX6, ZO1 and (**e**) TBR2 and KI67 at day 16 of neuronal differentiation in Mutant and two Corrected lines of brother 2. 3 independent experiments were performed with similar results. **f**, Rosette count in Mutant (M) and Corrected lines (C1 and C2) (top) and qPCR analysis for *TBR2* and *TUJI* mRNAs at day 16 in the second brother. Data are represented as mean ± SD of 3 independent experiments. The *p*-values by two-tailed unpaired Student’s t-test are indicated. *P* < 0.05 was considered statistically significant. **g**, Immunofluorescence for TBR2 and (**h**) CTIP2 and TUJI at day 32 of neuronal differentiation in Mutant and two Corrected lines of brother 2. Cells were counterstained with Hoechst. 3 independent experiments were performed with similar results. **i**, qPCR analysis for *TBR2, TUJI and CTIP2* mRNAs at day 32 in the second brother. Data are represented as mean ± SD of 3 independent experiments. The *p*-values by two-tailed unpaired Student’s t-test are indicated. *P* < 0.05 was considered statistically significant. **j**, Immunofluorescence for TBR2 and (**k**) CTIP2 and TUJI at day 60 of neuronal differentiation in Mutant and two Corrected lines of brother 2. Cells were counterstained with Hoechst. 3 independent experiments were performed with similar results. **l**, qPCR analysis for *TBR2, TUJI* and *CTIP2* mRNAs at day 60 in the second brother. Data are represented as mean ± SD of 3 independent experiments. The *p*-values by two-tailed unpaired Student’s t-test are indicated. *P* < 0.05 was considered statistically significant. **m**, Western blotting of nuclear and cytoplasmatic fractions in Mutant and Corrected lines of brother 2 at day 7, 14 and 30 of neuronal differentiation. GAPDH and Histone H3 were used to mark the cytosolic and nuclear fraction respectively. β-Catenin expression in the cytoplasm and nucleus is indicated. At day 14, 3 independent experiments, and at day 7 and day 30, 2 independent experiments were performed. GAPDH, Histone H3 and β-Catenin were run on the same gel. For gel source data see Supplementary Fig. 1. **n**, q-PCR analysis of *TBR2, CTIP2* and *TUJI* mRNAs at day 30 of neuronal differentiation after treatment regime according to Fig. 3e with the Wnt inhibitor IWP2 in Mutant cells and Wnt induction with recombinant Wnt3a in Corrected cells of brother 2. Data are represented as mean ± SD of 3 independent experiments. The *p*-values by two-tailed unpaired Student’s t-test are indicated. *P* < 0.05 was considered statistically significant. Abbreviations: M=Mutant; C1 and C2=Corrected 1 and Corrected 2.

In contrast to the substantial neurogenesis observed in the corrected clones, mutant cells expressed significantly reduced numbers of TUJI^+^ and CTIP2^+^ cells, as assessed by immunofluorescence microscopy (Fig. 1c) and quantitative PCR (qPCR; Fig. 1e, bottom, Extended Data Fig. 2k and Supplementary Tables 1 and 2). Extensive neuronal differentiation in mutant cells, as defined by the presence of TBR2^+^, CTIP2^+^ and TUJI^+^ cells, did not initiate until day 60 (Extended Data Fig. 3a-c). However, although markers for the early-born (CTIP2^+^) and late-born (SATB2^+^) neurons were found in both mutant and corrected cultures at days 60 and 90 of differentiation, the magnitude of their expression varied at the end points (Extended Data Fig. 3d-f), which suggested that the mutant cells maintained the ability to express all these cell types, but at a significantly delayed time scale. These phenotypic differences were confirmed in cells obtained from the second brother (Extended Data Fig. 4a-l).

We next asked whether the developmental delay in neurogenesis can also be found in vivo. We first determined the numbers of PAX6^+^ radial glial cells in the developing cortex of mice at embryonic day 13.5 (E13.5) and found no significant difference between wild-type (WT) and *Kdm5c* KO mice (Fig. 1f,i). The ventricular zone appeared similar in size (Fig. 1f, yellow lines); however, we observed a significantly reduced subventricular zone and cortical plate (Fig. 1g, yellow lines), which led to an overall reduced cortical thickness in the brains from *Kdm5c* KO mice. Consistent with the data from the patient-derived cells, we found a significant reduction in intermediate progenitors (TBR2^+^ cells) in the developing cortex of *Kdm5c* KO mice (Fig. 1g,j). The expression of the layer V/VI markers CTIP2 and TBR1 were also reduced (Fig. 1g,h,k,l), similar to that of the human iPS cell lines (Fig. 1a-e). Collectively, these data suggest that KDM5C regulates similar neurodevelopmental processes in mice and humans.

## Transcriptomic dysregulation in mutant cells

**Extended Data Fig. 5 ∣:**
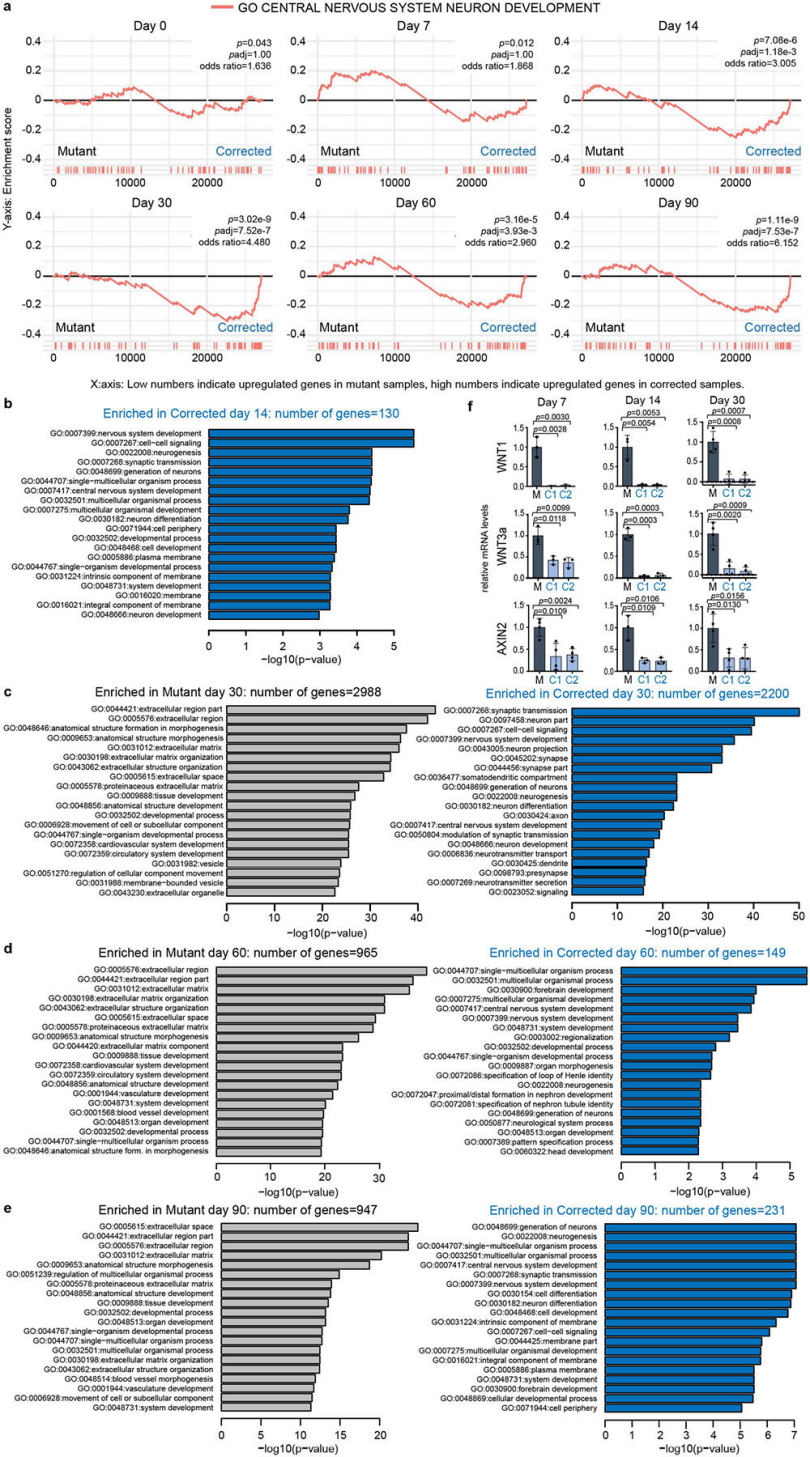
GO enrichment analysis in patient Mutant and Corrected cells. **a**, Gene Set Enrichment Analysis (GSEA) plots showing the enrichment of up and downregulated genes in the set of genes annotated as GO “central nervous system neuron development”. Genes are ordered based on the differential expression values obtained from DESeq2. *P*-values were calculated using Fisher’s exact test (two-sided). Exact *p*-values and multiple testing adjusted *p*-values are indicated. **b**, GO enrichment analysis showing GO terms that are upregulated in Mutant samples (enriched in Mutant) and downregulated in Mutant samples (enriched in Corrected) at day 14, (**c**) day 30, (**d**) day 60 and (**e**) day 90. **b-e**: *P*-values were calculated using goseq and adjusted for multiple testing. **f**, qPCR analysis of *WNT1, WNT3a* and *AXIN2* mRNAs at day 7 (left), 14 (middle) and 30 (right) of neuronal differentiation in Mutant and Corrected lines confirms RNA-seq data. Data are represented as mean ± SD of 3 (WNT1 day 7 and day 14, WNT3a day 7 and day 14 and AXIN2 day 14) and as mean ± SD of 4 (AXIN2 day 7 and WNT1, WNT3a and AXIN2 day 30) independent experiments. The *p*-values by two-tailed unpaired Student’s t-test are indicated. *P* < 0.05 was considered statistically significant.

**Extended Data Fig. 6 ∣:**
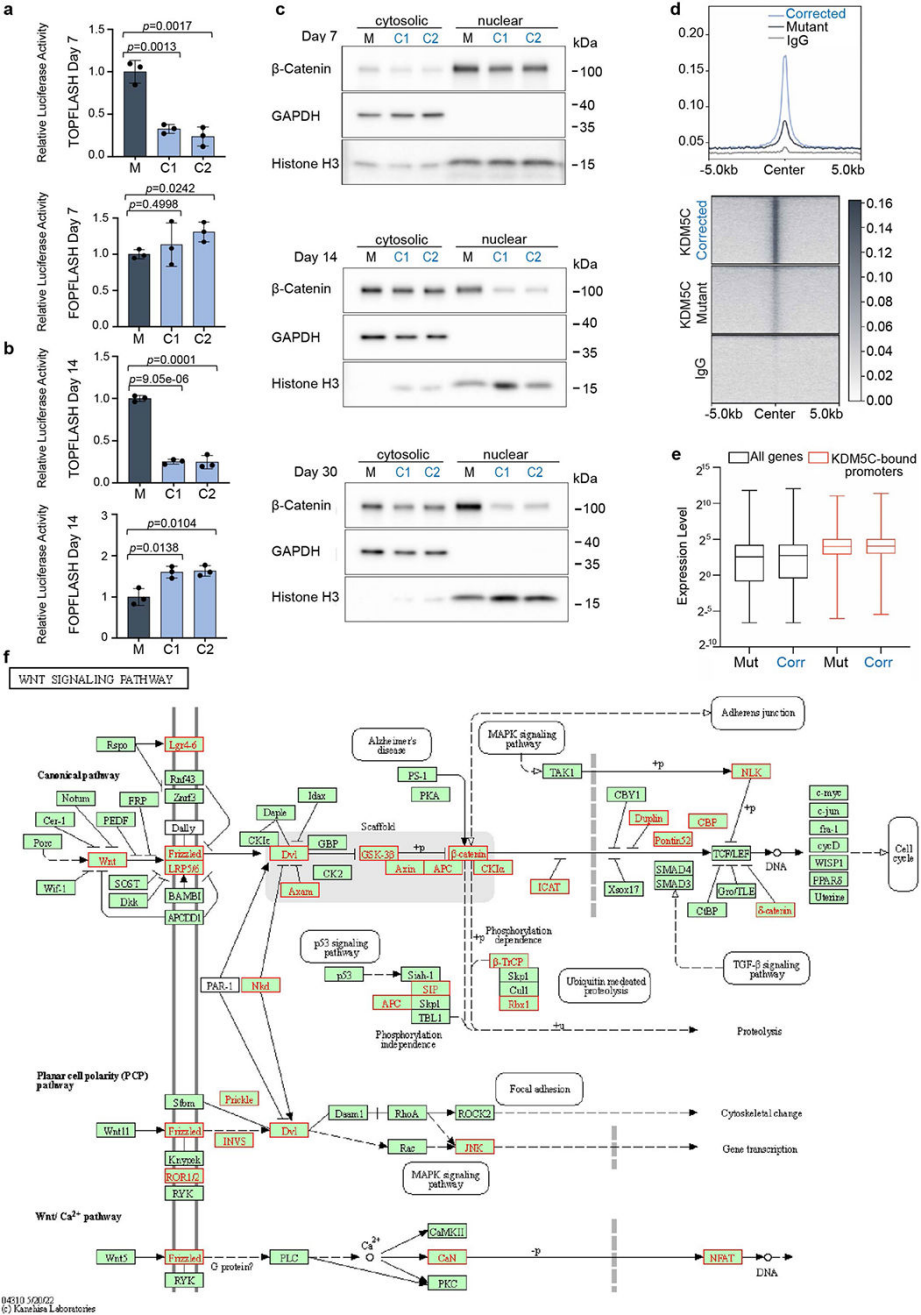
KDM5C binds directly to Wnt signaling genes. **a**, The relative luciferase activity in Mutant and Corrected cells (C1 and C2) transfected with Top-flash and Fop-flash vectors at day 7 and (**b**) day 14 of neuronal differentiation. Data are represented as mean ± SD of 3 independent experiments. The *p*-values by two-tailed unpaired Student’s t-test are indicated. *P* < 0.05 was considered statistically significant. Abbreviations: M=Mutant; C1 and C2=Corrected 1 and Corrected 2. **c**, Western blotting of nuclear and cytoplasmatic fractions isolated from Mutant and Corrected lines of brother 1 at day 7, 14 and 30 of neuronal differentiation. GAPDH and Histone H3 were used to mark the cytosolic and nuclear fraction respectively. β-Catenin expression in the cytoplasm and nucleus is indicated. 3 independent experiments were performed with similar results. GAPDH, Histone H3 and β-Catenin were run on the same gel. For gel source data see Supplementary Fig. 1. **d**, Profile plot and heat map of KDM5C enrichment at the 4,526 KDM5C peaks called in Corrected cells. **e**, Box plot representing gene expression levels in Mutant (Mut) and Corrected (Corr) cells for all genes and KDM5C bound promoters. Bounds of box indicate 25^th^ and 75^th^ percentiles. Centre line denotes median and bounds of whiskers indicate minima and maxima values. **f**, KEGG pathway highlighting genes in the Wnt signaling pathway directly bound by KDM5C (KEGG (hsa04310)). Genes directly bound by KDM5C are marked in red.

**Fig. 2 ∣:**
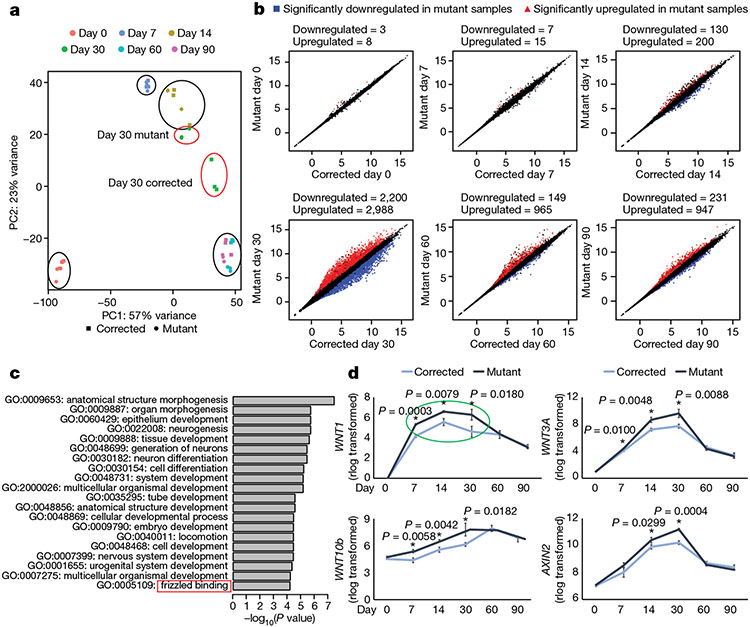
Transcriptome profiling and GSEA during neuronal differentiation. **a**, PCA of gene expression data from mutant and corrected cells using the top 1,000 genes with the highest variance across samples. Samples collected from different days during the 90-day differentiation protocol are indicated with different colours. Day 30 samples are highlighted with red circles. **b**, Scatter plots of normalized (rlog transformed) gene expression estimates for days 0, 7, 14, 30, 60 and 90. The numbers of significantly downregulated or upregulated genes are indicated on the top (log_2_ (fold change) ≥ 1, *P* ≤ 0.05). **c**, GO enrichment analysis showing genes that are upregulated in mutant cells at day 14. *P* values were obtained using goseq and adjusted for multiple testing. Red box highlights the GO term ‘frizzled binding’. **d**, rlog-transformed expression (*y* axis) levels of *WNT1, WNT3A, WNT10B* and *AXIN2* extracted from RNA-seq data at the indicated days (*x* axis) in mutant and corrected cells. Data represent the mean ± s.d. of three independent experiments. *P* values were calculated using two-tailed unpaired Student’s *t*-test; *P* < 0.05 was considered significant.

To gain insights into the molecular mechanisms that underlie KDM5C function, we performed a detailed RNA sequencing (RNA-seq) analysis of mutant and corrected cells at days 0, 7, 14, 30, 60 and 90 of neuronal differentiation in vitro. Principal component analysis (PCA) using the top 1,000 most variable genes showed that samples were separated based on time. Notably, the day-30 mutant sample clustered with the day-14 corrected sample, which indicated that mutant cells exhibit a differentiation delay at the transcriptomic level (Fig. 2a). This result is consistent with the differentiation delay we identified at the cellular level (Fig. 1). Significant expression differences emerged at day 14 and persisted throughout the analysis, and day 30 was transcriptionally the most divergent time point. Although day-60 and day-90 samples showed a reduced number of differentially expressed genes in comparison to day 30, gene expression differences were still apparent between the mutant and the corrected cells (Fig. 2b). Both gene set enrichment analysis (GSEA) and gene ontology (GO) over-representation analysis showed enrichment for nervous-system-related gene sets, such as neurogenesis, neuron projection and neurotransmitter secretion in corrected cells (Extended Data Fig. 5a-e). The GO term ‘frizzled binding’ was enriched in the mutant cells at day 14 of differentiation (Fig. 2c). Indeed, many crucial WNT–β-catenin pathway genes, including *WNT1, WNT3A, WNT10B* and the WNT–β-catenin target gene *AXIN2* (Fig. 2d), were significantly upregulated. Moreover, WNT genes belonged to the earliest (day 7) misregulated genes to emerge in our dataset (Fig. 2d and Extended Data Fig. 5f) and displayed a highly specific pattern. Specifically, day 30 showed the strongest misregulation in the mutant cells and no significant differences at the end points of differentiation (days 60 and 90; Fig. 2d, green circle). WNT activity reporter (TOP/FOPFlash) and β-catenin western blot analyses confirmed an increase in WNT activity at days 7, 14 and 30 of neuronal differentiation (Extended Data Figs. 4m and 6a-c). These results indicate that KDM5C is essential for directing the neurodevelopmental program and highlights a specific developmental window of WNT signalling misregulation in the mutant cells that correlates with the major phenotypic differences.

## KDM5C binds regulatory regions of WNT genes

**Fig. 3 ∣:**
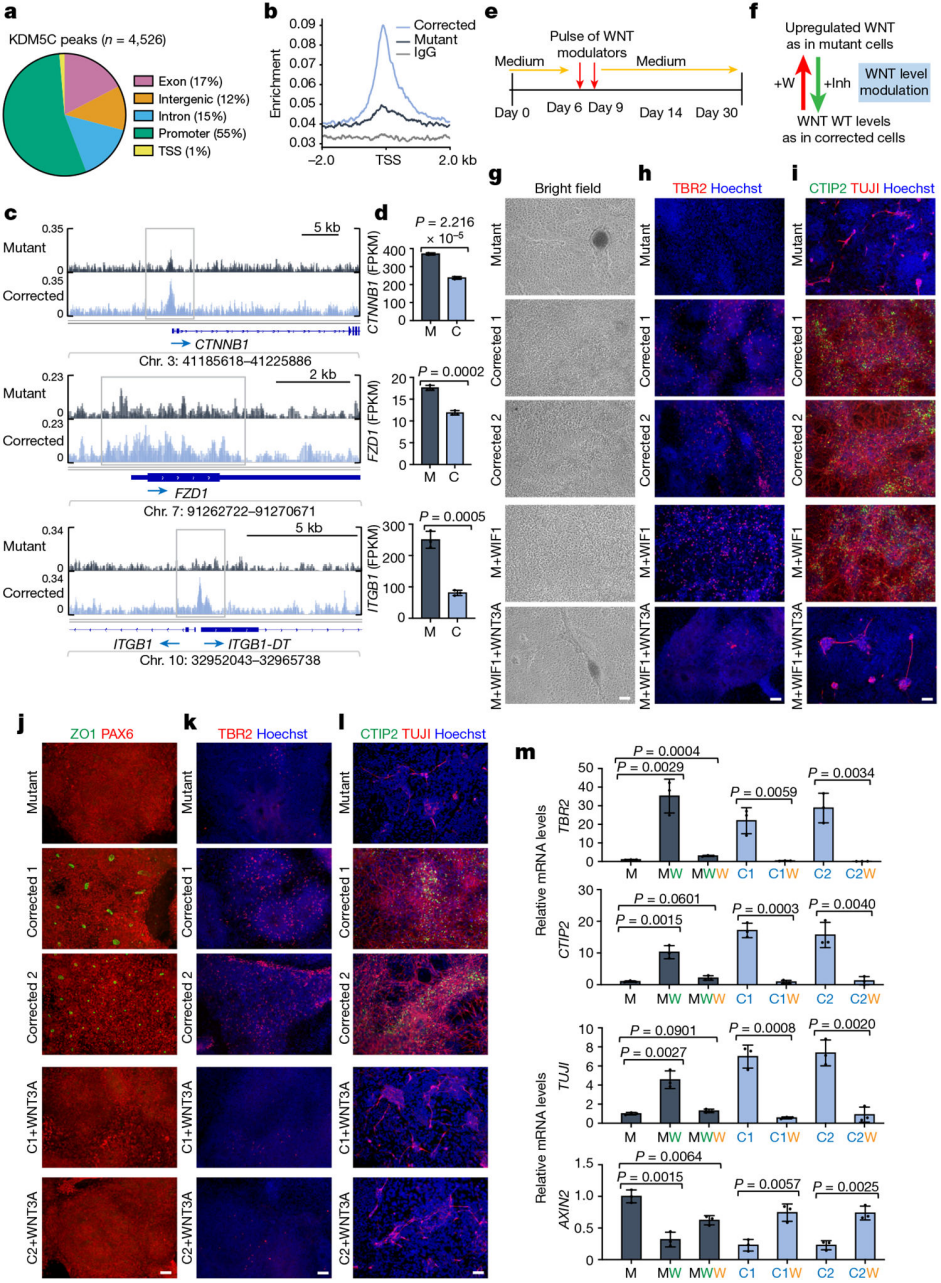
Delayed neuronal differentiation was mimicked or rescued by transient WNT–β-catenin modulation. **a**, Genomic distribution of KDM5C-binding events in corrected cells (CUT&RUN analysis on day 16). TSS, transcription start site. **b**, KDM5C enrichment profiles at promoter regions of genes. **c**, Integrative genomic viewer (IGV) snapshot of KDM5C-binding peaks at WNT-associated genes. **d**, Fragments per kilobase of transcript per million fragments mapped (FPKM) expression values extracted from RNA-seq data at day 16 for the presented WNT-associated genes. **e**, Scheme of the treatment strategy. Compounds were added on day 6 and day 9. On day 12, cells were washed and supplied with standard medium (Medium) that does not contain compounds. **f**, Schematic of the goal of treatment. Addition of WNT3A (+W) to corrected cells aimed to mimic WNT levels observed in mutant cells, surpassing WT levels that were considered optimal. Addition of a WNT inhibitor (+Inh) sought to reduce WNT to WT levels in mutant cells. **g–i**, Brightfield images (**g**) and immunofluorescence images of TBR2 (**h**) and for CTIP2 and TUJI (**i**) in mutant cells and corrected cells at day 30, which were treated transiently with vehicle (mutant (M), corrected 1 and corrected 2), WIF1 (1 μg ml^−1^; M+WIF1) or a combination of WIF1 (1 μg ml^−1^) and WNT3A (200 ng ml^−1^) (M+WIF1+WNT3A). **j–l**, Immunofluorescence analyses at day 30 of ZO1 and PAX6 (**j**), TBR2 (**k**) and CTIP2 and TUJI (**l**) and Hoechst. Corrected clones (C1 and C2) were transiently treated with recombinant WNT3A. **m**, qPCR analyses of *TBR2, CTIP2, TUJI* and *AXIN2* mRNA levels at day 30. Green W, WIF1; orange W, WNT3A. For **d** and **m**, data represent the mean ± s.d. of three independent experiments. *P* values were calculated using two-tailed unpaired Student’s *t*-test; *P* < 0.05 was considered significant. For **g–l**, more than three independent experiments were performed with similar results. Scale bars, 100 μm (**g–l**).

To identify direct target genes of KDM5C, we mapped KDM5C-binding sites throughout the genome in the mutant and corrected cells at day 16. We obtained 4,526 high-confidence KDM5C peaks in the corrected cells (2 out of 2 biological replicates; Fig. 3a). Substantially fewer (576) peaks were identified in the mutant cells, with an overall reduced KDM5C enrichment across all peaks (Extended Data Fig. 6d). KDM5C peak annotation in corrected cells at day 16 revealed a strong preference for promoter regions (near transcriptional start sites), which make up 55% of all KDM5C-enriched sequences (Fig. 3a,b). Additionally, genes bound by KDM5C were more highly expressed than average (Extended Data Fig. 6e), which suggested that KDM5C may be recruited to dynamically control their expression.

Notably, major components of the WNT–β-catenin pathway genes, including *CTNNB1* (which encodes β-catenin) and *FZD1,* are direct targets of KDM5C (Fig. 3c) and were upregulated in mutant cells (Fig. 3d). KDM5C also bound to the promoters of genes that encode extracellular matrix proteins such as *ITGB1* and *ITGB1-DT* (Fig. 3c,d), which have been implicated in cancer progression^17^. Analysis of all KDM5C peaks in the KEGG pathway database (hsa04310) further identified not only a strong association with the canonical WNT signalling pathway but also non-canonical WNT outputs (Extended Data Fig. 6f, gene names and boxes in red), which suggested that KDM5C directly regulates both canonical and non-canonical WNT pathways.

## Transient WNT–β-catenin modulation rescues mutants

The WNT signalling pathway has important roles in cortical neurogenesis, but its precise mode of action during neurodevelopment is complex and incompletely understood^18^. WNT can either promote self-renewal or differentiation of neuronal progenitor cells depending on their developmental stages^19-21^. Therefore, the early increased expression of WNT–β-catenin pathway genes (Fig. 2d and Extended Data Fig. 5f) in mutant cells and the direct association of KDM5C with crucial WNT signalling pathway genes (Fig. 3c and Extended Data Fig. 6f) could have an important mechanistic role in their differentiation delay.

To test this hypothesis, we developed a specific strategy of treatment (Fig. 3e) that consisted of two incubation pulses of WNT inhibitors at days 6 and 9 of neuronal differentiation. This treatment was designed to transiently downregulate WNT–β-catenin activity early during neuronal differentiation but allow WNT–β-catenin signalling activity later to mimic the expression and timing of WNT in the corrected cells (Figs. 2d and 3f). Consistent with our proposal that WNT–β-catenin overactivation is a key driver of pathologies in KDM5C mutant cells, treatment of such cells with two pulses of WNT inhibitory factor 1 (WIF1)^22^ during early differentiation was sufficient to restore the formation of intermediate progenitors (TBR2^+^) and early-born neurons (CTIP2^+^ and TUJ1^+^) to levels indistinguishable from corrected cells (Fig. 3g-i, compare the fourth panel with the second and third panels). Blocking WNT with inhibitor of WNT production 2 (IWP2), which works through an independent mechanism^23^, was similarly able to rescue the differentiation delay in mutant cells (Extended Data Fig. 3g,h). Furthermore, WNT inhibition in mutant cells restored the expression of neuronal markers (*TBR2, TUJ1* and *CTIP2*) to levels comparable with those in corrected cells (Fig. 3m and Extended Data Fig. 4n). However, when treatment was delayed for more than 14 days, no rescue of the phenotype was observed (data not shown). In support of the specificity of WNT inhibition, the phenotypes associated with both WNT inhibitors were suppressed by simultaneous addition of recombinant WNT3A (Fig. 3g-i, bottom, and Extended Data Fig. 3g,h, bottom). WNT–β-catenin upregulation was also sufficient to cause a neuronal differentiation delay, as corrected cells treated with recombinant WNT3A blocked rosette formation (Fig. 3j) and reduced the expression of differentiation markers (*TBR2, TUJ1* and *CTIP2*) to levels comparable to those in mutant cells (Fig. 3k-m). We therefore conclude that inappropriate upregulation of WNT activity in the identified developmental stage is necessary and sufficient for the delayed neuronal differentiation in *KDM5C* mutant cells.

## Reprogramming of the transcriptome and chromatin

**Extended Data Fig. 7 ∣:**
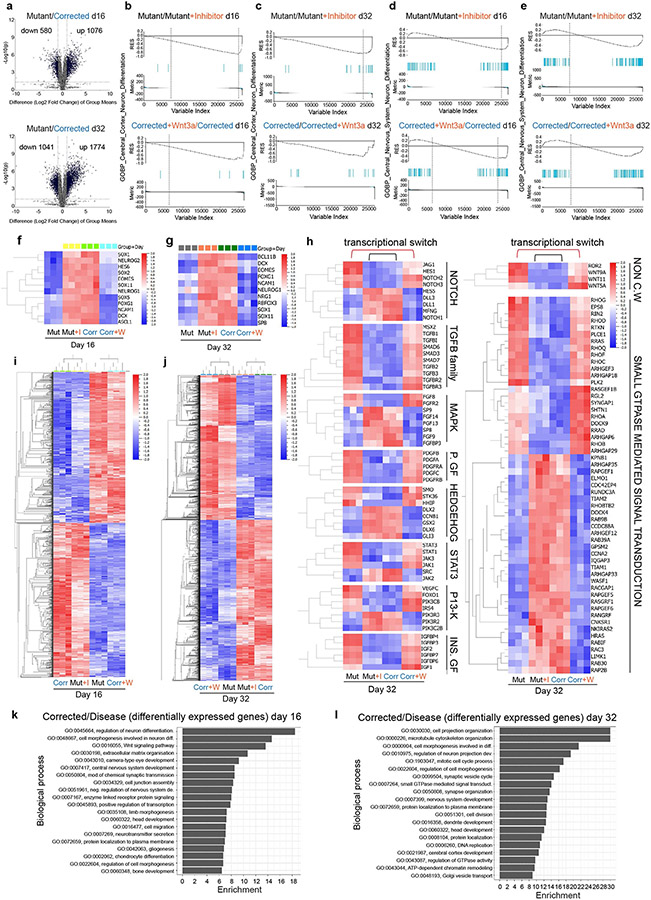
Transient modulation of the Wnt/β-catenin signaling pathway reprograms the transcriptomic landscape. **a**, Volcano plot showing differential gene expression (log2 fold change ≥ 1) between patient Mutant and Corrected cells at day 16 (top) and day 32 (bottom). *P*-values for a two-group comparison were calculated using a two-sided Student’s t-test. **b**, Gene Set Enrichment Analysis (GSEA) plots showing the enrichment of up and downregulated genes in the set of genes annotated as GO “Cerebral Cortex Neuron Differentiation” at day 16 and (**c**) day 32. Mutant versus Mutant+Inhibitor IWP2 (1 mM (first pulse)/0.25 mM (second pulse)) are shown at the top and Corrected versus Corrected+Wnt3a (200 ng/ml) are shown at the bottom. Genes are ordered based on the differential expression values obtained from DESeq2. **d**, Gene Set Enrichment Analysis (GSEA) plots showing the enrichment of up and downregulated genes in the set of genes annotated as GO “Central Nervous System Neuron Differentiation” at day 16 and (**e**) day 32. Mutant versus Mutant+Inhibitor (IWP2) are shown at the top and Corrected versus Corrected+Wnt3a are shown at the bottom. Genes are ordered based on the differential expression values obtained from DESeq2. **f**, Heatmaps showing gene expression for critical neuronal genes in Mutant (Mut), Mutant+Inhibitor (IWP2) (Mut+I), Corrected (Corr) and Corrected+Wnt3a (Corr+W) at day 16 and (**g**) day 32. **h**, Heatmaps for differential gene expression (q = 0.01) of transcripts for signaling pathways, growth factors (GF) and genes that are members of the GO enrichment term “small GTPase mediated signal transduction” at day 32 of differentiation that are sensitive to Wnt signaling perturbations. Abbreviations: PDGF = PDGF growth factors; INS=insulin; NON.C.W= non canonical WNT. **i**, Heatmaps for differential gene expression (q = 0.01) (log2fold change, q = 0.01) of transcripts between the disease group (Mutant and Corrected+Wnt3a) and the Corrected group (Corrected and Mutant+Inhibitor (IWP2)) at day 16 and (**j**) day 32 of neuronal differentiation. **k**, Gene ontology (GO) analysis of genes differentially expressed (q ≤ 0.01) between the Corrected group (Corrected and Mutant+Inhibitor) and the disease group (Mutant and Corrected+Wnt3a) at day 16 (top) and (**l**) day 32 (bottom). Extracted from **i** and **j**.

**Fig. 4 ∣:**
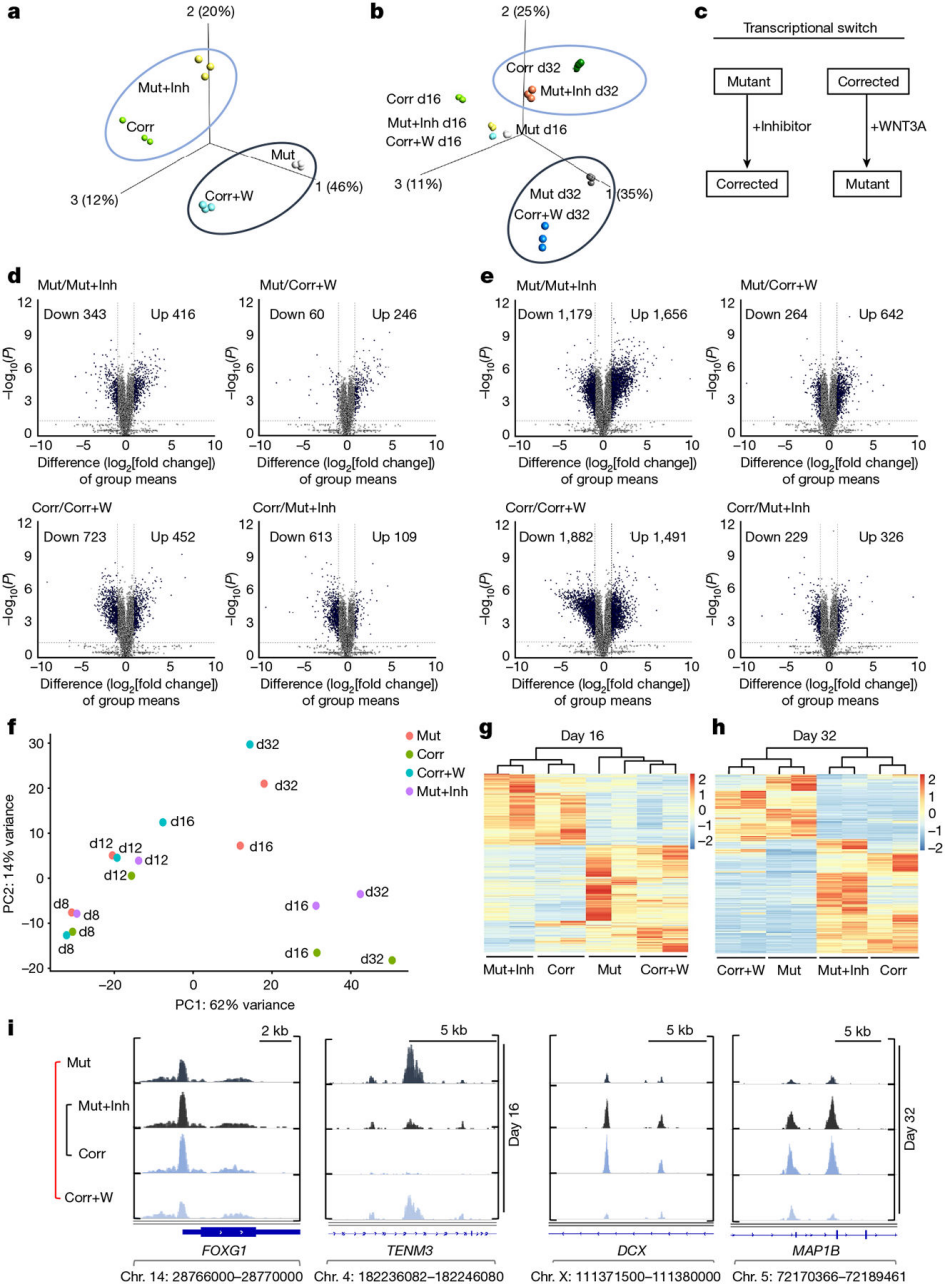
Transient modulation of the WNT–β-catenin signalling pathway reprograms the transcriptomic and chromatin landscapes. **a,b**, PCA plots of gene expression data from mutant cells (Mut), mutant cells treated with the WNT inhibitor IWP2 (1 μM (first pulse) and then 0.25 μM (second pulse)) (Mut+Inh), corrected cells (Corr) and corrected cells treated with WNT3A (200 ng ml^−1^) (Corr+W) using all 26,830 genes (iGenome UCSC hg38) in our dataset at day 16 (**a**) and day 32 (d32) (day 16 (d16) results also included) (**b**) of neuronal differentiation. **c**, Mutant cells exhibit a global transcriptomic change, referred to as reprogramming, towards the profile of corrected cells after brief WNT inhibitor treatment. Conversely, corrected cells undergo transcriptional reprogramming to mirror the mutant cell transcriptome following transient treatment with recombinant WNT3A protein. **d,e**, Volcano plots at day 16 (**d**) and day 32 (**e**) (log_2_(fold change) ≥ 1 and *P* ≤ 0.01) for transcripts detected by RNA-seq analysis. Top, mutant lines are compared with either mutant + inhibitor cells (left) or corrected + WNT3A cells (right). Bottom, corrected cells are compared either to corrected + WNT3A cells or mutant + inhibitor cells. Cut-off at *P* value = 0.05 and log_2_ (fold change) = ±1. *P* values for two-group comparison were calculated using two-sided Student’s *t*-test. **f**, PCA of ATAC–seq data from mutant cells, mutant cells treated with the WNT inhibitor IWP2 (1 μM (first pulse) then 0.25 μM (second pulse)), corrected cells and corrected cells treated with WNT3A (200 ng ml^−1^) using all peaks in the genome at days 8, 12, 16 and 32 of neuronal differentiation. **g,h**, Heatmaps representing open chromatin regions (false discovery rate (FDR) ≤ 0.05) between the indicated cell lines at day 16 (**g**) and day 32 (**h**). **i**, IGV snapshot of open chromatin peaks (ATAC–seq) between the indicated cell lines at days 16 and 32 of differentiation.

In our analyses discussed above, we investigated the impact of transient WNT–β-catenin manipulation only on specific neuronal markers (Fig. 3m) but not the whole transcriptomic landscape. We therefore used the same treatment regimen (Fig. 3e) and performed RNA-seq analyses at day 16 and day 32 of differentiation (Extended Data Fig. 7a). PCA using all genes revealed that the mutant cells, when treated transiently with the WNT inhibitor, diverged significantly from the untreated mutant cells and became similar to the corrected cells (Fig. 4a-c). The same phenomenon was observed when corrected cells were transiently treated with WNT3A. Instead of grouping with the corrected cells with which they share the genomic origin, they clustered with the mutant cells (Fig. 4a,b).

We next investigated the degree of transcriptome changes induced by the WNT inhibitor in mutant cells and by the WNT3A recombinant protein in corrected cells. We compared their gene expression levels with those of untreated cells of the same genetic background (that is, mutant compared with mutant plus inhibitor) and with phenotypically similar counterparts (that is, mutant compared with corrected plus WNT3A). We observed clear differences at day 16 (Fig. 4d) and at day 32 (Fig. 4e). The difference between mutant and mutant plus inhibitor became substantial at day 32, with 1,656 and 1,179 upregulated and downregulated genes, respectively, compared with 642 and 264 upregulated and downregulated genes, respectively, when mutant was compared with corrected plus WNT3A (Fig. 4e, top left and right). A similar phenomenon was observed when we compared corrected cells with the treated cell lines. At day 16 and day 32, the comparison between corrected and corrected plus WNT3A showed a much higher divergence than the comparison between corrected versus mutant plus inhibitor (Fig. 4d,e, bottom). These findings suggest that the global transcriptomic landscape in mutant cells can be restored through short-term WNT inhibition to resemble that of corrected cells. In addition, the disease-associated transcriptome observed in mutant cells can be induced in corrected cells through transient WNT upregulation. Together, these results confirm that the WNT–β-catenin pathway is a major downstream effector of KDM5C.

GSEA showed that genes upregulated in the mutant plus inhibitor condition (but not in the mutant cells) were enriched for neurodevelopmental genes at day 16 and day 32 of differentiation (Extended Data Fig. 7b-e, top). By contrast, corrected cells treated with WNT3A lost their enrichment for neuronal genes essential for proper neuronal differentiation dynamics (Extended Data Fig. 7b-e, bottom). Notably, a panel of major neuronal regulator genes (Extended Data Fig. 7f,g) and genes associated with different aspects of the signalling landscape (Extended Data Fig. 7h) were highly affected by transient WNT perturbations.

To understand the functional differences between these treatments, we performed GO annotation. On day 16 (Extended Data Fig. 7i,k) and day 32 (Extended Data Fig. 7j,l), the analysis substantiated the enrichment of GO terms associated with neuronal differentiation and WNT signalling. It also revealed an enrichment of GO terms related to cell projection organization, microtubule cytoskeleton organization, regulation of neuron projection, synapse organization and dendrite development (Extended Data Fig. 7k,l). The high level of enrichment of genes related to the GO term ‘small GTPase mediated signal transduction’ suggests a strong association between transient WNT–β-catenin perturbations and their expression (Extended Data Fig. 7h, right).

**Extended Data Fig. 8 ∣:**
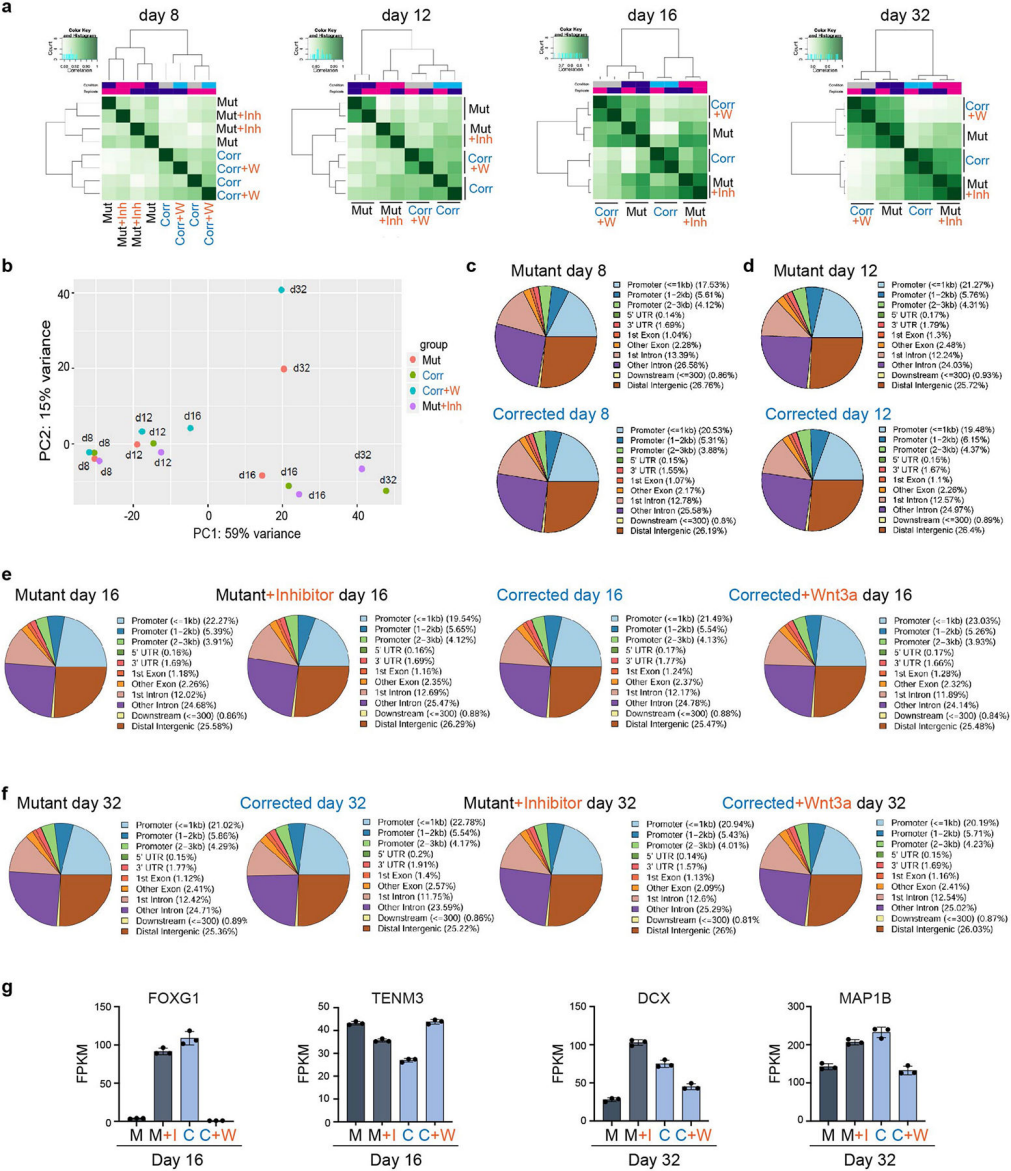
Modulation of the Wnt/β-catenin signaling pathway reprograms the chromatin landscape. **a**, Correlation heatmap of open chromatin regions (i.e. ATAC-seq peaks) at day 8, 12, 16 and 32. **b**, Principal component analysis (PCA) of ATAC-seq signals using all peaks in the genome at day 8, 12, 16 and 32 of neuronal differentiation representing all samples from the second biological replicate. **c**, Pie charts showing the genomic distribution of open chromatin regions (i.e. ATAC-seq peaks) in patient Mutant and Corrected cells at day 8 and (**d**) day 12. **e**, Pie charts showing the genomic distributions of open chromatin regions in Mutant, Mutant+Inhibitor (IWP2) (1 mM (first pulse)/0.25 mM (second pulse)), Corrected and Corrected+Wnt3a (200 ng/ml) cells at day 16 and (**f**) day 32. **g**, FPKM expression values for selected genes as extracted from RNA-seq data at day 16 and day 32. Data are represented as mean ± SD of 3 independent experiments. Abbreviations in the Figure: M or Mut=Mutant; C or Corr=Corrected; I or Inh=Wnt Inhibitor; W=Wnt3a.

We then investigated the dynamics of chromatin accessibility in mutant and corrected cells, as well as in response to transient WNT pathway activation (corrected plus WNT3A) and inhibition (mutant plus inhibitor). We used ATAC–seq and focused our analysis on the time window between treatment initiation (day 8) and day 32 of differentiation, a time point at which we observed the most prominent differences in transcription. At day 12, cells started to diverge, and changes in chromatin organization became apparent at day 16 and day 32 when mutant cells were clustered with corrected plus WNT3A cells, and corrected cells were clustered with mutant plus inhibitor cells (Fig. 4f-h and Extended Data Fig. 8a,b). Comparing all cell lines between day 8 and day 32, we found no significant difference in the distribution of peaks throughout the genome (Extended Data Fig. 8c-f).

**Extended Data Fig. 9 ∣:**
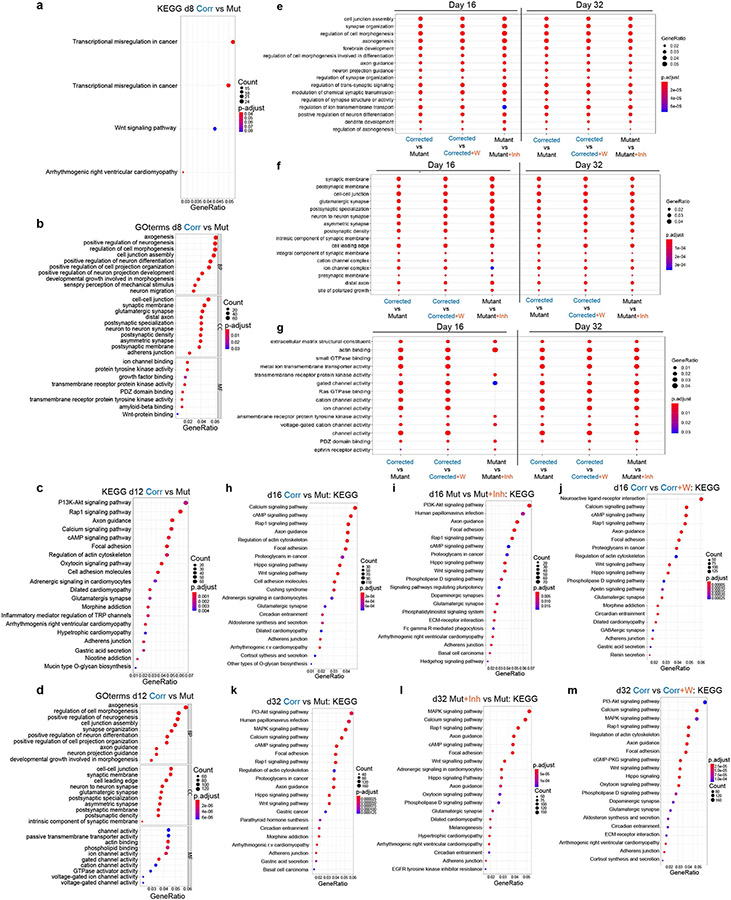
Transient modulation of the Wnt/β-catenin signaling pathway reprograms the chromatin landscape. **a** and **b**, KEGG and Gene ontology (GO) analysis in patient Mutant and Corrected cells at day 8 and **c** and **d**, day 12. Abbreviations: BP=Biological Processes; CC=Cellular Components; MF=Molecular Function. **e**, Most enriched Gene ontology (GO) terms for Biological Process, **f**, Cellular Components and **g**, Molecular Function for differentially accessible chromatin regions between three cellular comparisons (Corrected versus Mutant, Corrected versus Corrected+Wnt3a (+ W) (200 ng/ml) and Mutant versus Mutant+Inhibitor (+ Inh) cells (IWP2, 1 mM (first pulse)/0.25 mM (second pulse)) at day 16 and day 32 (FDR ≤ 0.05). **h-j**, KEGG analysis at day 16 and, **k-m**, day 32 between the same three cellular comparisons as in **e-g** (FDR ≤ 0.05). **a-m**, One-sided Fisher exact tests with multiple comparison correction were performed.

Next, we aimed to determine whether there is an overlap in the functional classifications that change between corrected and mutant cells when comparing corrected cells to those treated with WNT3A, and mutant cells to those treated with a WNT inhibitor. As shown in Extended Data Fig. 9e-g, all the top GO terms were identical and showed similar gene ratios. The identified GO and KEGG terms overlapped with our RNA-seq data (Figs. 2 and 4), representing terms related to brain development, dendrite development and small GTPase binding (Extended Data Fig. 9). The enrichment for these gene classes were of particular interest because structural differences in synapse formation and dendrite development are hallmarks of the adult brains of *Kdm5c* KO mice and individuals with ID^13,24^. Representative profiles demonstrating changing chromatin accessibility at promoters of genes associated with spine density and dendrite formation are shown in Fig. 4i and Extended Data Fig. 8g. In summary, this global impact on the transcriptomic and chromatin landscapes was notable considering the short duration of the treatment (Fig. 3e). Moreover, at day 32 of differentiation, cells were already more than 2 weeks without any treatment, which emphasizes the long-term impact of transient modulation of the WNT–β-catenin pathway on the transcriptomic and chromatin landscapes.

## WNT manipulation in vivo affects mouse behaviour

**Extended Data Fig. 10 ∣:**
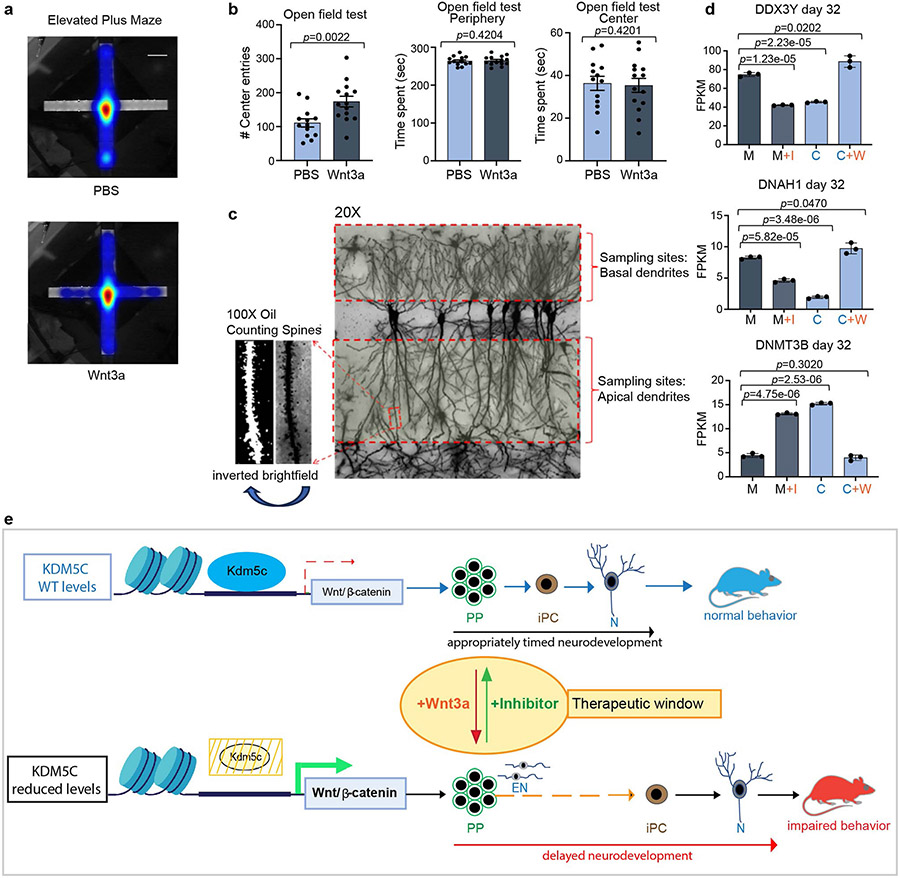
A single Wnt3a injection induces and Wnt signaling inhibition rescues *KDM5C*-associated ID phenotypes, respectively. **a**, Representative heatmaps in the Elevated Plus Maze for PBS and Wnt3a injected mice. **b**, Number of entries into the center in the Open field test (left), time spent in the periphery (middle) and the center (right) in the Open field test. Data are represented as mean ± SEM. 13 PBS and 14 Wnt3a injected mice were investigated. The *p*-values by one-tailed unequal variance t-test are indicated. *P* < 0.05 was considered statistically significant. **c**, Animation of sampling sites for spine density analysis provided by Neurodigitech. **d**, Expression of the germ line genes DDX3Y, DNAH1 and the DNA methyltransferase DNMT3b at day 32 in the indicated cell lines extracted from RNA-seq data. Data are represented as mean ± SD of 3 independent experiments. The *p*-values by two-tailed unpaired Student’s t-test are indicated. *P* < 0.05 was considered statistically significant. **e**, Model: KDM5C does not efficiently bind to Wnt signaling genes in Mutant cells, which have significantly reduced KDM5C levels. Subsequently, early premature neurons (EN) are briefly increased but more importantly the transition from primary progenitor cells (PP) to intermediate progenitors (iPC) is delayed, which results in a significant delay of neuron (N) expression and proper inter-neuronal connectivity with reduced spine density. During this particular time period of neuronal development, Wnt sensitivity is elevated such that only transient Wnt perturbation is sufficient to have either therapeutic effects when Wnt is inhibited in Mutant cells or can induce disease phenotypes when Wnt3a is added to Corrected cells resulting in persistent cognitive impairments in adult mice. Model was in part created with BioRender.com.

**Fig. 5 ∣:**
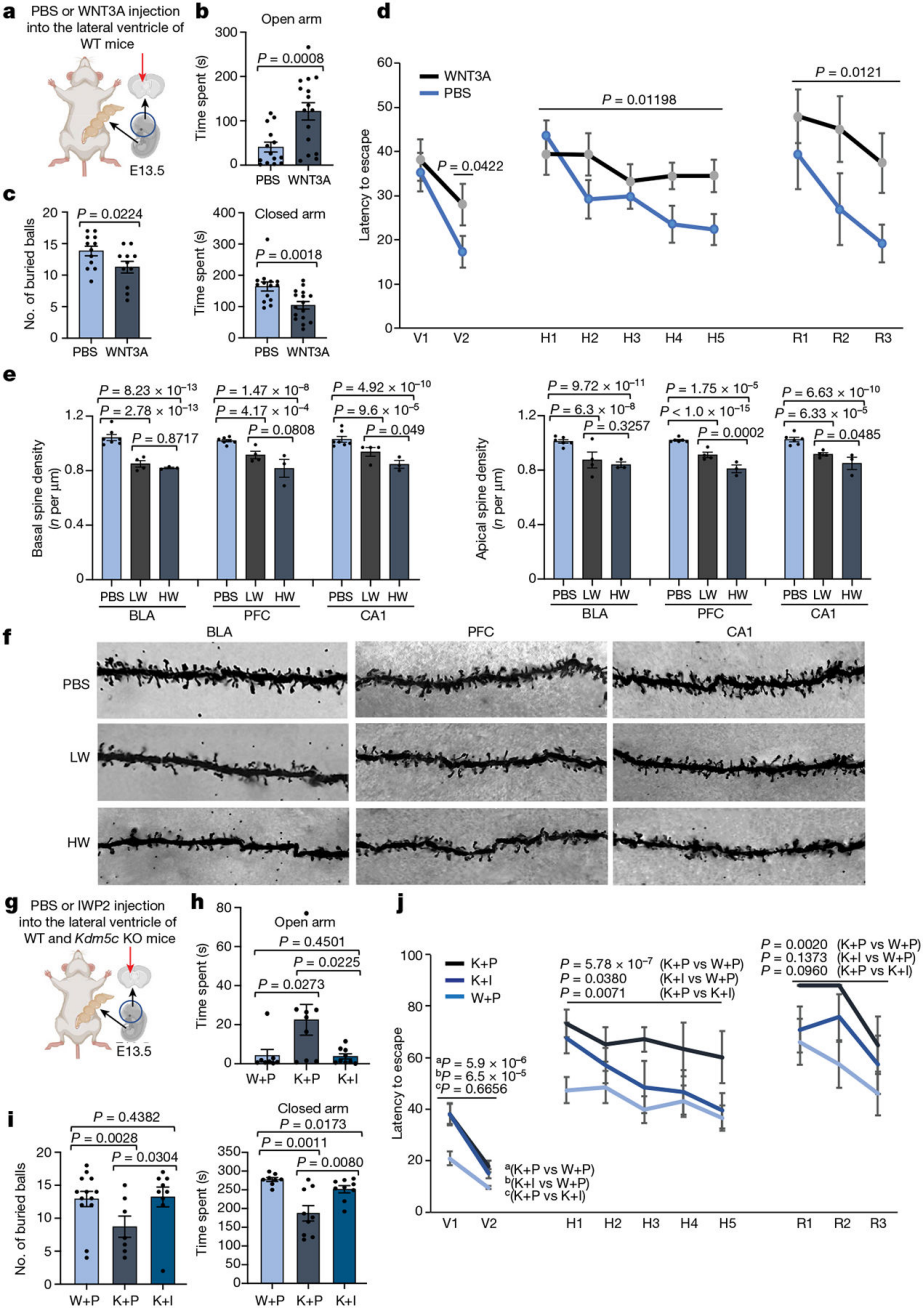
A single WNT3A injection induces and WNT inhibition rescues *KDM5C*-associated ID phenotypes. **a**, Schematic of the experiment. PBS or recombinant WNT3A (50 ng) was injected into the lateral ventricle of WT E13.5 embryos. **b**, Elevated-plus maze anxiety test. *n* = 14 (PBS treated) and *n* = 16 (WNT3A treated) mice. **c**, Marble-burying test in PBS-injected mice (*n* = 12) and WNT3A-injected mice (*n* = 11). Data for **b** and **c** represent the mean ± s.e.m. *P* values calculated using one-tailed unequal variance *t*-test. **d**, Morris water maze escape latency test. One-tailed unequal variance *t*-test for results from the visible platform condition (V1 and V2) and two-way analysis of variance (ANOVA) for the hidden platform (H1–H5) and reversal (R1–R3) conditions were performed. *P* values are indicated. Data are presented as the mean ± s.e.m. *n* = 15 (PBS injected) and *n* = 17 (WNT3A injected) mice. **e**, At E13.5, WT embryos were injected with PBS, a low dose of WNT3A (33 ng; LW) or a high dose of WNT3A (50 ng; HW). Spine density in the basolateral amygdala (BLA), prefrontal cortex (PFC) and CA1 of the hippocampus was analysed (number of spines per μm). One-way ANOVA followed by Tukey’s multiple comparison test is presented based on single measurements averaged for each group. *P* values are indicated. Data are expressed as the mean ± s.e.m. of average values for each mouse. *n* = 7 (PBS) mice for basal spine density, *n* = 6 (PBS) mice for apical spine density, *n* = 4 mice for LW treatment and *n* = 3 mice for HW treatment. **f**, Representative dendritic segments from BLA, PFC, and CA1 regions in mice treated with PBS or with a high or low dose of WNT3A. **g**, Schematic of injections in E13.5 WT (PBS) and *Kdm5c* KO embryos (PBS or the WNT inhibitor IWP2 (9.34 ng)). **h**, Elevated plus maze anxiety test. Number of mice analysed: 8 WT + PBS (W+P), 9 KO + PBS (K+P) and 9 KO + IWP2 (K+I). **i**, Marble-burying test. Number of mice analysed: 13 WT + PBS, 7 KO + PBS and 9 KO + IWP2. Data for **h** and **i** are presented as the mean ± s.e.m. *P* values were calculated using one-tailed unequal variance *t*-test. **j**, Morris water maze escape latency test. Two-way ANOVA for V1–V2, H1–H5 and R1–R3 was performed. *P* values are indicated. Data are expressed as the mean ± s.e.m. *n* = 12 WT + PBS, 7 KO + PBS and 9 KO + IWP2 mice. *P* < 0.05 was considered significant. Details provided in the Methods. Illustrations in **a** and **g** were created using BioRender (https://www.biorender.com).

To determine whether transient WNT–β-catenin pathway upregulation during development in vivo is sufficient to induce behavioural changes in WT adult mice, akin to those in *Kdm5c* KO mice, we injected recombinant WNT3A or PBS (as control) into the lateral brain ventricle of E13.5 embryos (Fig. 5a). This period marks the time point when radial glial cells begin to produce substantial numbers of intermediate progenitors (TBR2^+^) similar to the specific window targeted during human iPS cell differentiation (Fig. 3e). Embryos were allowed to develop to term and were analysed 4–6 months after birth. WNT3A-treated mice showed reduced anxiety levels based on the elevated-plus maze paradigm and marble-burying test (Fig. 5b,c and Extended Data Fig. 10a), which indicated that transient WNT–β-catenin activation during embryonic brain development mimics the ID caused by *KDM5C* mutations. In the open-field test, we observed a significant difference in the number of entries into the centre between PBS-treated mice and WNT3A-treated mice (Extended Data Fig. 10b, left). The total time spent in the centre or in the periphery was not significantly different (Extended Data Fig. 10b, middle and right). This finding is consistent with two previous independent studies^13,14^ showing that *Kdm5c* KO mice exhibit reduced anxiety in the elevated-plus maze and marble-burying test but not in the open-field test. Results from Morris water maze tests showed a significant difference in the ability of PBS-treated mice and WNT3A-treated mice to find the visible or hidden platforms as well as during the reversal phase (Fig. 5d). This result indicated that WNT3A treatment is also sufficient to compromise spatial-learning formation. No gross abnormalities in the cytoarchitecture or the expression of layer-specific markers were found in adult *Kdm5c* KO mice^13^. Therefore, the gene expression changes observed during early neurodevelopment may have impaired spine development and density, which are not only hallmarks of adult *Kdm5c* KO brains^13^ but also a hallmark of many human neurodevelopmental disorders^25^. Consequently, we investigated whether behavioural alterations in adult mice are associated with changes in spine density. We administered intraventricular injections of WNT3A at E13.5 (Fig. 5a) and examined three brain regions (the prefrontal cortex, the basolateral amygdala and CA1 of the hippocampus), all of which could contribute to the observed behavioural alterations. In all three regions, we observed a reduction in spine density in both basal and apical dendrites of pyramidal cells (Fig. 5e,f and Extended Data Fig. 10c). Notably, we also found dose-dependent changes in spine density that were mostly evident in the basal and apical dendrites of the hippocampus CA1 and the apical dendrites of the prefrontal cortex (Fig. 5e). These findings demonstrate that brief WNT manipulation during embryonic brain development alters dendritic spine complexity and density in the adult mouse brain. This result aligns with an enhanced accessibility of regulatory regions controlling genes crucial for spine density regulation in response to treatment of mutant cells with the WNT inhibitor (Fig. 4i) and could underlie the behavioural phenotypes observed in both *Kdm5c* KO mice and individuals with *KDM5C*-mediated ID.

Last, we asked whether the behavioural alterations in anxiety and memory formation in *Kdm5c* KO male mice could be rescued by a transient reduction in WNT signalling during this developmental time window. PBS or the WNT inhibitor IWP2 was injected into the lateral ventricle of E13.5 embryos and the mice were analysed between 4 and 7 months of age. A single injection of IWP2 was sufficient to significantly rescue the behaviours of *Kdm5c* KO mice. That is, the cognitive abilities of the treated mice were more like those of WT mice, as shown in results from the elevated-plus maze and marble-burying test. The Morris water maze test also showed that the treated mice had improvements in learning and memory (Fig. 5g-j).

In summary, our data show that transient WNT–β-catenin manipulation during the neurodevelopmental window identified in this study is sufficient to cause (WT mice) or to rescue the ID-related behavioural phenotypes associated with *Kdm5c* KO mice. These findings support our hypothesis that WNT–β-catenin misregulation is a major cause of *KDM5C* mutation-associated symptoms in humans (Extended Data Fig. 10e).

## Discussion

Neurodevelopment involves the precise temporal and spatial formation of progenitors and neurons^2-4^. Although the consequences of sustained disruption in the developmental sequence are well documented, little is known about how neurodevelopmental delays may affect the formation of cortical circuits and cognition^2,26^. Here we identified KDM5C, a potential central hub of a multi-component pathogenic cascade involving other related neurodevelopmental disorder genes^27^, as an essential safeguard to control the precise timing of the neurodevelopmental sequence. In particular, we discovered that significant KDM5C reduction in patient-derived cells is associated with an inefficient and delayed entry of primary progenitors into the intermediate progenitor stage and consequently into neurons^28^, and this function of KDM5C seemed to be conserved between humans and mice (Fig. 1). Mechanistically, we demonstrated that KDM5C directly targets the canonical WNT signalling pathway, thereby identifying WNT as a major downstream effector of KDM5C and a potential therapeutic target for interventions in ID. Although downstream factors of WNT signalling are well explored, our understanding of the upstream factors remains incomplete. Our findings showed that KDM5C is an important upstream regulator of the WNT pathway during development. Given the involvement of WNT in tumorigenesis, our data may have additional implications for cancer research, particularly considering the role of KDM5C as a tumour suppressor in certain cancers^29^.

Importantly, previous well-designed studies of WNT–β-catenin signalling focused on long-term alterations^19-21^ and were not necessarily designed to show the enduring effects of temporary WNT activity modulation during neurodevelopment. Our treatment strategies, however, were explicitly crafted for this purpose. This effort revealed a long-lasting impact of transient WNT modulations on the global transcriptomic and epigenomic landscapes of human mutant and corrected cells. Specifically, substantial numbers of important cortical and signalling genes were tightly controlled by WNT–β-catenin activity (Extended Data Fig. 7f-h). In addition, several non-canonical WNT members seemed to be controlled by WNT3A (Extended Data Fig. 7h), which suggests that changes in phenotypes may also include β-catenin-independent WNT signalling. Additionally, KDM5C, an upstream regulator of WNT, directly binds the promoters of these genes (Extended Data Fig. 6f). In this context, the large numbers of misregulated genes associated with small GTPase signal transduction are worth noting (Extended Data Fig. 7h, right), as members of the small GTPase family play crucial parts during neurodevelopment and have been implicated in essential neuronal functions such as migration, dendrite development and axonal extension^30^. This finding is of particular interest because the phenotypes observed for adult *Kdm5c* KO mice and in *Drosophila* studies showed a reduction in dendritic branching and length as well as in spine density and formation^13,31,32^, but the origin of these defects and the precise mechanisms were thus far unknown. Our data revealed that a brief WNT manipulation substantially affected the transcriptional programs necessary for cortical circuit formation (Fig. 4 and Extended Data Figs. 7-9), with effects on spine formation and density later in life. This result suggests that the changes in spine morphology associated with KDM5C loss are probably a result of misregulated WNT activity during embryonic neurodevelopment. Given that this effect of WNT is dose-dependent, it is possible that spine density is tuneable by manipulating WNT levels, which can have important clinical implications.

Among the noteworthy misregulated genes in the *KDM5C* mutant cells that are controlled by WNT–β-catenin modulation is *FOXG1* (Extended Data Fig. 8g), a master regulator of brain development^33^. As individuals carrying mutations in *FOXG1* and *KDM5C* share phenotypic similarities^33^, FOXG1 may have an important role downstream of KDM5C and the canonical WNT signalling pathway. Additionally, recent mouse studies revealed that KDM5C contributes to the developmental silencing of germline genes in collaboration with an unknown cofactor^14^. Consistent with these findings, we observed that the germline genes *DNAH1* and *DXX3Y* were not silent in mutant cells and showed an inverse correlation with the de novo DNA methyltransferase DNMT3B (Extended Data Fig. 10d). This result suggests that proper activation of the canonical WNT signalling pathway could be an essential factor in suppressing germline genes during neurodevelopment^14^.

To assess whether dysregulation in the developmental program results in persistent cognitive impairments in adult mice, we injected recombinant WNT3A into the lateral brain ventricle of E13.5 WT embryos. These investigations were based on the challenging hypothesis that a singular event at the appropriate developmental time may have enduring effects on cognition. So far, therapeutic effects in adult animal models have only been achieved with chronic WNT treatments. Once treatment was aborted, disrupted cognitive defects returned^34^. Previous studies showed that treatment with Lithium (LiCl) or GSK3 inhibitors, which lead to activation of the WNT–β-catenin signalling pathway, improves cognitive impairments in various animal models. Here we not only observed an opposite role of WNT–β-catenin signalling upregulation—with cognitive impairment rather than improvement—but we also identified a specific developmental window in which persistent cognitive alterations can be induced when WNT–β-catenin signalling was modulated. This result highlights the particular importance of the timing of WNT intervention at the developmental stage when radial glial cells switch from symmetric to asymmetric division to produce intermediate progenitor cells, an apparently sensitive period during neurodevelopment in which only a small shift in the balance of WNT expression levels has long-term consequences on cognition. So far, no treatments for *KDM5C* mutation-associated ID are available as no specific therapeutic targets or window of clinical perturbations have been identified. In this context, it is worth noting that in clinical trials, adults with fragile X syndrome, which shares many cognitive phenotypes with *KDM5C* mutation-associated ID, treated with lithium showed only minor improvements^35,36^. A possible explanation for this result is that the intervention may have occurred at a suboptimal developmental time. Our data substantiates this notion, as we demonstrated that cognitive phenotypes induced by KDM5C loss can be ameliorated with a single injection of a WNT inhibitor during the identified developmental period (Fig. 5). In this context, our study provides a conceptual and experimental framework for systematically investigating the roles of WNT, potentially in a variety of neurological conditions and beyond, and the potential impacts of transient WNT modulation conducted in an appropriate developmental window.

## Methods

### Reprogramming of patient-derived fibroblasts

Patient-derived fibroblasts were cultured in DMEM (Life Technologies, 11995073) supplemented with 10% FBS (Invitrogen, 12676011), 2 mM l-glutamine (Corning, 25-005-CI), 1×MEM non-essential amino acids (Invitrogen, 11140050) and 100 U ml^−1^ penicillin–streptomycin (Corning, 30-002-CI).

Reprogramming was performed using non-integrating episomal plasmid vectors^37^. In brief, 800,000 fibroblasts were washed in PBS and then resuspended in Amaxa nucleofector solution (Nucleofector kit for human dermal fibroblast; VPD-1001) with plasmids encoding OCT4, SOX2, KLF4, LMYC and LIN28A together with *TP53* shRNA (Addgene, 27077, 27078 and 27080). Cells were treated with Program P22 of an Amaxa Nucleofector 2 device. Cells were plated onto a well of a 6-well plate and cultured for 6 days in fibroblast medium. On day 6, cells were dissociated in 0.05% trypsin, transferred onto 0.1% gelatin-coated 10 cm plates on irradiated mouse embryonic fibroblast (MEF) feeder cells CF-1 (GlobalStem, GSC-6201G) and cultured for 14 days in DMEM/F12 and 15 mM HEPES (StemCell Technologies, 36254) supplemented with 20% KnockOut serum replacement (Invitrogen, 10828-028), 100 mM 2-mercaptoethanol (Gibco, 21985-023), 2 mM l-glutamine (Corning, 25-005-CI), 1×MEM non-essential amino acids (Invitrogen, 11140050) and 10 ng ml^−1^ bFGF (Bio Pioneer, HRP-0011-1). On day 21, smooth compact colonies were manually picked onto 24-well plates and expanded. Human iPS cell lines were investigated for expression of pluripotency markers (immunofluorescence and qPCR), lack of *EBNA1* DNA integration and normal karyotype^37,38^.

### Generation of isogenic controls

We used CRISPR technologies to generate double-strand breaks at the mutation site to increase the frequency of homologous recombination. Simultaneously, we provided a correction template for homologous recombination that utilizes PiggyBac technology to seamlessly correct the mutation. The correction template contains arms of homology for the region, corrects the mutation and introduces a drug-selection cassette flanked by repeats recognized by PiggyBac transposase. Once iPS cell lines that have undergone homologous recombination have been selected and identified, the selection cassette was removed using a hyperactive form of the PiggyBac transposase. A detailed description of the methodology has been previously published^39^.

### Human iPS cell culture

Human iPS cells were cultured on irradiated MEF feeder cells CF-1 (GlobalStem, GSC-6201G) in DMEM/F12 and 15 mM HEPES (StemCell Technologies, 36254) supplemented with 20% KnockOut serum replacement (Invitrogen, 10828-028), 100 mM 2-mercaptoethanol (Gibco, 21985-023), 2 mM l-glutamine (Corning, 25-005-CI), 1×MEM non-essential amino acids (Invitrogen, 11140050) and 10 ng ml^−1^ bFGF (Bio Pioneer, HRP-0011-1). Cells were fed and inspected daily, and differentiated cells at the edges of colonies were manually removed. Cells were passaged either manually or with collagenase (Invitrogen, 17104-019). iPS cells were routinely karyotyped and tested for mycoplasma contamination.

### Neuronal differentiation

Before neuronal differentiation was started, cells were inspected daily and differentiated cells were manually removed. At least two passages before differentiation, cells were cultured at the same densities and colony size. On the day of differentiation, differentiated cells were removed and MEFs were lifted from the cultures. The time of manipulation between each line was kept identical because the timing of preparation influences the general cell state and the plating efficiency. During the cleaning and preparation stage, cells were cultured in MEF-conditioned medium supplemented with Y-27632 (Selleck Chemicals, S1049) at 10 μM. The conditioned medium containing Y-27632 was added to all cultures at and for the exact same time. The neuronal differentiation protocol was performed according to a previously published method^40^ with modifications designed by V.K.-N., which gave excellent reproducibility.

The neuronal maintenance medium^40^ comprised a 1:1 mixture of N-2 (Gibco, 17502048) and B-27 (Gibco, A35828-01). N-2 medium consists of DMEM/F-12 (Fisher Scientific, mt10092cv), 1× N-2, 5 μg ml^−1^ insulin (Sigma-Aldrich, I9278-5M), 1 mM l-glutamine (Corning, 25-005-CL), 100 μm non-essential amino acids (Invitrogen, 11140050), 100 μM 2-mercaptoethanol (Gibco, 21985-023), 50 U ml^−1^ penicillin and 50 mg ml^−1^ streptomycin (Corning, 30-002-CL). B-27 medium consists of neurobasal (Life Technologies, 21103-049), 1× B-27, 200 mM l-glutamine, 50 U ml^−1^ penicillin and 50 mg ml^−1^ streptomycin.

The neuronal induction medium^40^ comprised neural maintenance medium supplemented with 1 μM dorsomorphin (Sigma-Aldrich, P5499-5MG) and 10 μM SB431542 (Selleck Chemicals, S1067).

### Neuronal differentiation in the context of rescue experiments

Two ways to perform the rescue experiment related to the data presented in the paper are described here. Cells were cultured until day 5 of differentiation in neural induction medium^40^. On day 5, cells were passaged using collagenase (Invitrogen, 17104-019) at a one-quarter ratio in neuronal induction medium. On the following day, once all cells settled down, the medium was changed to neuronal induction medium supplemented with vehicle, WIF1 (R&D Systems, 1341-WF-050, final concentration of 1 μg ml^−1^), IWP2 (Sigma-Aldrich, I0536-5MG) or WNT3A (R&D Systems, 5036-WN-010/CF; final concentration of 200 ng ml^−1^). The medium volume was calculated for 3 days. After the first incubation, cells were washed 2 times with neuronal induction medium. Cells were then replated in neuronal induction medium overnight and were incubated again with the compounds at the same concentrations but in neuronal maintenance medium for the subsequent 3 days. In total, compounds were added only twice into the medium for the entire time of treatment. After the incubation time, cultures were washed once in PBS and twice in neuronal maintenance medium and cultured according to the protocol in standard neuronal medium (neuronal maintenance medium).

As IWP2 is an effective compound, it can be used at high cell densities. In this case, cells do not need to be passaged at day 5 or 6 but can be maintained until day 9 with a supplementation of IWP2 at a concentration of 1 μM to the neuronal induction medium from day 6 to day 9 (only added once early at day 6). After this period of time, cells were washed and replated in neuronal induction medium. On the following day, medium was changed to neuronal maintenance medium as described above and cells were cultured in these conditions for an additional day. Thereafter, cells were treated for a further 3 days with 0.25 μM of IWP2 (only added once to the culture with no daily supplementation) in neuronal maintenance medium. The same regimen was performed for WIF1. In this case WIF1 concentrations were increased to adapt to the higher cell densities and therefore higher WNT ligand production.

### Immunostaining iPS cells

Human iPS cells were carefully washed with 1×PBS and fixed with 4% paraformaldehyde (PFA) for 15 min at room temperature. PFA was washed out 3 × 10 min. Permeabilization was performed with 0.1% Triton X-100 in PBS for 5 min and cells were blocked with 3% serum (the same as the secondary antibody), 1% BSA in PBS 0.1% Triton X-100 at room temperature for at least 1 h. Incubation with primary antibodies diluted in blocking solutions was performed overnight at 4 °C. Cells were washed 3 × 10 min in PBS, and secondary antibodies were incubated in blocking solution for 1 h. Secondary antibodies were used at 1:1,000 dilutions (Alexa Fluor, Invitrogen). Cells were counterstained with DAPI or Hoechst (indicated in figures). The antibodies used in this study are shown in Supplementary Table 1.

### Immunostaining E13.5 embryos

*Kdm5c* WT and *Kdm5c* KO^13^ E13.5 male littermates were decapitated, and whole heads were washed twice in 1×PBS and fixed with 4% PFA overnight. After incubation, heads were washed 3 × 10 min in 1×PBS and incubated in 20% sucrose overnight. The next day, heads were embedded in OCT and snap-frozen by immersion in 2-methylbutane cooled on dry ice. Cryosection was performed using a Leica CM3050 S Cryostat. Serial 14 μm coronal sections were made throughout the whole cortex and slides were mounted onto charged SuperFrost Plus slides. To ensure complete collection of cortical tissue, no trimming was performed. The total number of labelled cells per fixed field per section was calculated. Results are expressed as the mean value of marker^+^ cells per field ± s.d. and were tested for significance using two-sided unpaired Student’s *t*-test. *P* < 0.05 was considered significant.

For immunostaining, slides were washed in PBS, permeabilized for 5 min in 0.04% Tween-20 in PBS and 5% serum and blocked for 2 h in the same solution. Slides were then incubated with primary antibodies diluted in 5% goat serum, 0.3% Triton X-100 in PBS overnight. The next day, primary antibodies were washed out with PBS (3 × 10 min) and incubated for 1 h at room temperature with secondary antibodies at 1:1,000 dilutions (Alexa Fluor, Invitrogen). Finally, slides were washed in PBS and coverslipped with Fluoromount-G (Southern Biotech). The antibodies used are shown in Supplementary Table 1.

Images were acquired using a Nikon Eclipse TE2000-U inverted fluorescence microscope.

### Western blot analysis

Whole cell lysates were prepared using RIPA buffer (150 mM NaCl, 1% Nonidet P-40, 0.5% sodium deoxycholate, 0.1% SDS and 25 mM Tris pH 7.4). Lysates were run on an 8% SDS–PAGE gel and transferred to nitrocellulose membranes (Bio-Rad, 1620112). Membranes were blocked for 1 h in 0.05% Tween and PBS (PBST) with 5% non-fat dry milk, then incubated overnight with primary antibody (1:1,000 dilution of primary antibody in PBST with 5% non-fat dry milk). The next day, membranes were washed (3 washes of 5 min each with 10 ml of PBST), incubated with HRP-conjugated secondary antibodies for 1 h in PBST with 5% non-fat dry milk, then washed again (3 washes of 5 min each with 10 ml of PBST). HRP-conjugated antibodies were detected using ECL western blotting detection reagents (PerkinElmer Western Lighting Plus-ECL, NEL104001 EA) according to the manufacturer’s instructions. Full scan blots are available in Supplementary Fig. 1.

### Subcellular fractionation and immunoblotting

Subcellular fractionation was performed using a previously published protocol^41^ with modifications. The following buffers were used: cytosolic fraction buffer: 150 mM NaCl (Invitrogen, AM9759), 50 mM Tris pH 7.5 (Invitrogen, 15567-027), 20 μg ml^−1^ digitonin (TCI, D0540); membrane fraction buffer: 150 mM NaCl, 50 mM Tris pH 7.5, 1% NP40 (Sigma, I3021); nuclear fraction buffer (RIPA): 150 mM NaCl, 50 mM Tris pH 7.5, 0.5% sodium deoxycholate (Sigma, D6750), 0.1% SDS (Fisher BioReagents, BP166-500) and 250 U ml^−1^ of Universal Nuclease for Cell Lysis (Pierce, 88701). Protease and phosphatase inhibitors (Pierce, A32961) were added immediately before use.

The entire process of subcellular fractionation was performed at 4 °C. Cells were washed with 1×PBS and incubated with cytosolic fraction buffer for 5 min. Cells were resuspended and spun down for 10 min at 2,000*g*. The supernatant was collected as the cytosol-enriched fraction. The pellet was washed once with 1×PBS and resuspended with membrane fraction buffer for 30 min. Cells were spun down for 10 min at 7,000*g*. The supernatant was collected as the membrane-enriched fraction. The pellet was washed once with 1×PBS and resuspended with nuclear fraction buffer for 1 h. Cells were vortexed and spun down for 10 min at 7,000*g*. The supernatant was collected as the nuclear-enriched fraction. Samples were denatured in Laemmli SDS sample buffer (Boston BioProducts, BP-111R) for 5 min at 97 °C, run on an SDS–PAGE and transferred to an Immobilon-P membrane (Millipore, IPVH85R). Membranes were blocked with 5% BSA (Jackson ImmunoResearch, 001-000-162) in TBST (Boston BioProducts, IBB-180). Primary and secondary antibodies (conjugated with HRP) were diluted in 5% BSA. Chemiluminescence was detected using ECL Prime western blotting detection reagent (Cytiva, RPN2236) on a ChemiDoc MP imaging system (Bio-Rad, 12003154). For samples from brother 1: 3 biological replicates were performed per day. For brother 2: day 14: *n* = 3 biological replicates; day 7 and day 30: *n* = 2 biological replicates. Full scan blots are available in Supplementary Fig. 1.

### Dual luciferase assay

Cells were plated in 48-well plates and transfected with 225 ng Super TOPFLASH (Addgene, 12457) or FOPFLASH (Addgene, 12456) and 4.5 ng EF1α-Renilla^42^. Cells were transfected in triplicate using Lipofectamine 3000 (Invitrogen, L3000015). Dual luciferase reporter assays were performed using a Dual-Luciferase Reporter assay system (Promega, E1960) according to the manufacturer’s instructions. Luminescence was measured using an EnSight Multimode plate reader (PerkinElmer). For clearer presentation, values from mutant cells were set to 1, and values for corrected clones were calculated to the mutant value = 1. Error bars for luciferase represent the s.d.; *n* = 3 biological replicates. Significance was determined using Student’s *t*-test (values are presented in the figure legends).

### RNA analysis

Total RNA was isolated using TRIzol reagent according to the manufacturer’s instructions (Life Technologies, 15596018). cDNA was synthesized using a PrimeScript RT reagent kit (Takara Bio, RR037Q) and qPCR analysis was performed on a LightCycler 480 (Roche) with cDNA equivalent to 200 ng total RNA. The SYBR Green (Roche, LightCycler480 SYBR Green I Master, 04887352001) protocol consisted of denaturation at 95 °C for 5 min followed by 45 cycles of 95 °C, 10 s; 60 °C, 10 s and single 72 °C with a single data acquisition during each extension cycle. Primers for qPCR are listed in Supplementary Table 2.

Gene expression was normalized to endogenous *GAPDH* expression, or the relative expression of the respective gene was determined after normalization to *GAPDH* and calculated using the following formula: relative expression level = 2ΔΔCT. Subsequently, for clearer presentation, values from mutant cells were set to 1, and values from corrected clones were calculated to mutant value = 1. All graphs containing the label ‘relative mRNA levels’ were calculated based on these considerations. Error bars in all figures represent the s.d.; *n* = 3–4 biological replicates (exact numbers are presented in the figure legends). Significance was determined using Student’s *t*-test (values are presented in figure legends). Graphs were generated using GraphPad Prism (v.10.0.3).

### RNA-seq library preparation

RNA library preparation for data presented in Fig. 2 and Extended Data Fig. 5 was performed using a NEBNext Ultra Directional RNA Library Prep kit for Illumina according to the manufacture’s guidelines (New England BioLabs, E7420L, E7490L and E7335L). Multiplexed libraries were pooled in equimolar ratios and were purified from a 1.5% TBE-agarose gel using a PureLink Quick Gel extraction kit (Invitrogen, K2100-12). The libraries were sequenced to a length of 50 bases using an Illumina HiSeq 2500, High Output v4 at the Tufts Genomics Core (Tufts University) according to standard procedures. Library quality was measured on a Bioanalyzer at the Tufts Genomics Core (Tufts University).

For RNA-seq, input RNA samples were first subjected to quality check using an Agilent Fragment Analyzer. Only RNA samples that passed quality control were then used for library preparation using an Illumina mRNA Sample Preparation kit per the manufacturer’s instructions. The molar concentrations of the resulting libraries were then quantified on the Fragment Analyzer, adjusted and mixed to equal molar mixture. The pooled libraries were sequenced on an Illumina NextSeq 550 using v.2.5 High Output chemistry and single-read 75 bases format. The base-calling and demultiplexing were performed on the raw data using Illumina bcl2fastq.

### CUT&RUN

CUT&RUN for KDM5C was performed according to a Cell Signaling Cut&Run Assay kit (86652) with modifications developed by V.K.-N. Libraries were performed using a NEBNext UltraII DNA Library prep kit for Illumina according to the manufacture’s guidelines (New England Biolabs, E7645, E7335 and E7500). Multiplexed libraries were pooled in equimolar ratios. Library quality was measured on a Bioanalyzer at the Molecular Genetics Core at Boston Children’s Hospital. The libraries were sequenced on an Illumina NextSeq 500 System using a NextSeq 500/550 High Output kit v2.5 (75 cycles) at the Molecular Genetics Core at Boston Children’s Hospital.

### ATAC–seq

Cryopreserved cells were sent to Active Motif. The cells were then thawed in a 37 °C water bath, pelleted, washed with cold PBS and tagmented as previously described^43^, with some modifications based on ref. 44. In brief, cell pellets were resuspended in lysis buffer, pelleted and tagmented using the enzyme and buffer provided in the ATAC–seq kit (Active Motif). Tagmented DNA was then purified using a MinElute PCR purification kit (Qiagen), amplified with 10 cycles of PCR and purified using Agencourt AMPure SPRI beads (Beckman Coulter). The resulting material was quantified using a KAPA Library Quantification kit for Illumina platforms (KAPA Biosystems) and sequenced with PE42 sequencing on a NextSeq 500 sequencer (Illumina).

### Bioinformatics

#### RNA-seq data.

RNA-seq data relating to Fig. 2 and Extended Data Fig. 5 were mapped against the human genome version hg19 with STAR (v.2.5.2b)^45^. R (v.3.4.1)^46^ and Bioconductor (v.3.6)^47^ were used for the RNA-seq analysis. Reads were counted using the R package GenomicAlignments (v.1.14.0)^48^ (mode=‘Union’, inter.feature=FALSE) and only primary read alignments were retained. rlog-transformed values of the counts and differential expression values were calculated using DESeq2 (v.1.18.0)^49^. Figure 2 was created using ggplot2 (v.2.2.1)^50^.

The GSEA was done according to a previously published method^51^. GO analysis results were prepared using the goseq (v.1.28.0) package^52^.

For the data relating to Fig. 4 and Extended Data Fig. 7, the resulting demultiplexed data were aligned to the human reference genome hg38 using HISAT (v2.1.0). Read count normalization (FPKM) and differential expression analysis were performed using Cufflinks (v.2.1.0). The resulting normalized count table was modified and used as input for visualization and GSEA using Qlucore Omics Explorer (v.3.8)^53,54^.

#### CUT&RUN data.

CUT&RUN reads (Fig. 3 and Extended Data Fig. 6) were mapped to hg38 using Bowtie (v.2.3.5.1), with the parameters --no-unal --local --very-sensitive-local --no-mixed --no-discordant --phred33 -I 10 -X 700. Bigwig files were generated using BamCoverage in Deeptools (v.3.3.1), with the parameters --binSize 20 -- normalizeUsing BPM. MACS (v.2.2.6) was used to call KDM5C peaks on each replicate individually, with the --nolambda parameter. Bedtools (v.2.28.0) was used to subtract blacklist regions (using bedtools subtract) and to identify peaks called in both replicates (using bedtools intersect). The blacklist file was downloaded from GitHub (https://github.com/Boyle-Lab/Blacklist/blob/master/lists/hg38-blacklist.v2.bed.gz). Peaks mapping to chrM and chrUn were removed, and peak annotation was performed using homer-4.11, with annotatePeaks.pl, and the fraction of peaks associated with each genomic feature was plotted in Prism 9. IGV version 2.11.9 was used to visualize data.

### ATAC–seq data

ATAC–seq data ATAC–seq analysis for data relating to Fig. 4f and Extended Data Fig. 8b was performed by Active Motif. Reads were aligned using the BWA algorithm (mem mode; default settings). Duplicate reads were removed, and only reads mapping as matched pairs and only uniquely mapped reads (mapping quality &gt=1) were used for further analysis. Alignments were extended in silico at their 3′ ends to a length of 200 bp and assigned to 32-nucleotide bins along the genome. The resulting histograms (genomic ‘signal maps’) were stored in bigWig files. Peaks were identified using the MACS (v.2.1.0) algorithm at a cut-off of *P* = 1 × 10^−7^, without control file, and with the–nomodel option. Peaks that were on the ENCODE blacklist of known false ChIP–seq peaks were removed. Signal maps and peak locations were used as input data to Active Motif’s proprietary analysis program, which creates Excel tables containing detailed information on sample comparison, peak metrics, peak locations and gene annotations.

The remaining analysis was performed by the Harvard Chan Bioinformatics Core. First, quality assessment of the ATAC–seq data was performed using FASTQC (v.0.11.8) (http://www.bioinformatics.babraham.ac.uk/projects/fastqc), and the data were processed using the ATAC-seq pipeline bcbio-nextgen (v.1.2.8) that includes the following steps. Reads were filtered and trimmed using Atropos (v.1.1.29)^55^. High-quality reads were mapped to the human genome (build GRCh38/hg38) using Bowtie2 (v.2.4.1)^56^. Mitochondrial DNA reads were filtered from the dataset, and properly paired reads with high mapping quality (MAPQ score > 10, non-duplicates, qualified reads) were retained using Sambamba (v0.7.1)^57^ for further analysis. The ‘alignmentSieve’ function of Deeptools (v.3.5.0)^58^ and ‘sort’ and ‘index’ functions of Samtools (v.1.9)^59^ were used to isolate fragments in nucleosome-free regions. Reads were shifted by 9 bp (+4 in positive and −5 in negative strand) to account for the dimeric binding of the Tn5 transposase that results in insertion of two adaptors separated by 9 bp. To call the peaks with unique reads, we used MACS2 (v.2.2.7.1)^60^. ATAC–seq data quality was assessed using ataqv (v.1.2.1)^61^. CPM-normalized bigwig files (bin size = 20) were visualized using IGV (v.2.8.4)^62^. Sets of peaks were compared using BEDTools (v.2.27.1)^63^. Statistical analysis was performed in R (v.3.6.1).

Differential accessibility was assessed using Diffbind (v.3.0.15) (http://bioconductor.org/packages/release/bioc/vignettes/DiffBind/inst/doc/DiffBind.pdf) with DESeq2 (v.1.30.1)^49^ and including batch in the model. Peaks were considered differentially enriched at FDR < 0.05. The genomic distribution of the peaks was annotated using ChIPseeker (v.1.26.2)^64^. Functional enrichment analysis was performed using ClusterProfiler (v.3.18.1)^65^.

### Animal studies

All experiments involving animals were conducted in accordance with the protocols approved by the Institutional Animal Care and Use Committee (IACUC) of Boston Children’s Hospital. All mice were housed in individually ventilated cages with a 12-h light–dark cycle and with ad libitum access to food and water. Mice were housed in temperatures of 18–24 °C with 40–60% humidity. *Kdm5c* KO mice were generated as previously described^13^.

#### In utero intracerebroventricular injections.

All in utero experiments were performed under protocols approved by the IACUC at Boston Children’s Hospital. Timed-pregnant CD-1 dams were obtained from Charles River Laboratories. At E13.5, dams were anaesthetized by isoflurane inhalation and laparotomy was performed. Recombinant WNT3A protein (R&D Systems, 5036-WN-010/CF, 50 ng (for behavioural studies) and 33 ng (for lower dosage spine density analyses)) and the WNT signalling inhibitor IWP2 (9.34 ng; Sigma Aldrich, 10536) were dissolved in PBS. A volume of 1 μl of PBS, WNT3A or IWP2 were injected very slowly into the lateral ventricle of E13.5 embryos using fine glass capillary pipettes (Drummond Scientific, 21-176-2C) as previously described^66^. Meloxicam analgesia was subcutaneously injected following surgery according to the IACUC protocol. All mice that developed a hydrocephalus or other injuries as a consequence of the surgeries were euthanized. To ensure that only healthy mice were part of our dataset, we also inspected mice brains after behavioural studies were completed.

### Behavioural studies

All behavioural testing was performed by the Animal Behavior and Physiology Core Facility at Boston Children’s Hospital. All investigators were blinded. Male and female mice were between 4 and 7 months old when testing was performed on WT mice that were injected with recombinant WNT3A or PBS. Rescue experiments were performed in *Kdm5c* KO male mice injected with IWP2 or PBS and in WT control male mice injected with PBS. Breeding for rescue experiments was performed as previously described^20^.

#### Marble-burying test.

Twenty glass marbles (Dark) (approximately 15 mm in diameter) were placed equidistant in a 4 × 5 arrangement. The light intensity in the cage was adjusted to 30 lux. The number of buried marbles after 30 min was measured. *n* = 12 PBS-treated mice and *n* = 11 WNT3A-treated mice were used for WNT3A induction experiments in WT mice. *n* = 7 KO + PBS mice, *n* = 9 KO + IWP2 mice and *n* = 13 WT + PBS mice were used for rescue experiments in *Kdm5c* KO mice and WT controls as indicated.

#### Open-field test.

For anxiety assessment, a mouse was placed in an arena of 40 × 40 cm in which the centre area measured an arena of 20 × 20 cm. The mouse explored the arena for 15 min (data were collected in 5-min bins). Video analysis and data acquisition were obtained using Noldus EthoVision XT video tracking software (v.15.0, Noldus Information Technologies). The time spent in the centre area (10 × 10 cm) and periphery was calculated as a measure of anxiety. *n* = 13 PBS-treated mice and *n* = 14 WNT3A-treated mice.

#### Elevated plus-maze test.

Anxiety behaviour was tested using the elevated plus-maze test. The maze (Med Associates) consisted of two open arms (35.5 × 6 cm) and two closed arms (35.5 × 6 cm) radiating from a central area (6 × 6 cm). A 0.5-cm-high lip surrounded the edges of the open arms, and 20-cm-high walls enclosed the closed arms. The arms were underlit with infrared light and mice were tracked and scored using Noldus Etho-Vision XT video tracking software (v.15.0, Noldus Information Technologies). *n* = 14 PBS-treated mice and *n* = 16 WNT3A-treated mice were used for WNT3A induction experiments in WT mice. *n* = 9 KO + PBS mice, *n* = 9 KO + IWP2 mice and *n* = 8 WT + PBS mice were used for rescue experiments in *Kdm5c* KO mice and WT controls as indicated

#### Morris water maze test.

A white, opaque, circular tub (60 cm depth × 83 cm diameter) was filled to 29 cm deep with water that was approximately 25 °C. Four visible, distinct shapes were placed in each of the four quadrants of the inner walls of the tub to form distinct quadrants. Trials were videotaped and scored using EthoVision XT video tracking software (v.12.0, Noldus Information Technologies). Acquisition training (day 1) consisted of 8 trials per mouse with a white platform (10 cm diameter) 0.5 cm above the surface of the water and marked with a red reflector. Each training trial began by lowering the mouse into the water close to the pool edge. The start location for each trial was alternated in a semi-random order for each mouse. Each mouse started in each of the quadrants twice. Hidden training (day 2–3) consisted of 20 trials per mouse (12 on day 2 and 8 on day 3) with the platform placed in a new quadrant, 1 cm below the water. Each mouse started in each of the quadrants five times. For reversal training, the platform was placed in a new quadrant, 1 cm below the water. Each mouse completed three trials, each starting from the position opposite of the new platform location. Mice were allowed a maximum of 90 s to reach the platform. During visible, hidden and reversal trials, a mouse that failed to reach the platform in 60 s was guided to the platform by the experimenter. Mice were left on the platform for 5 s before being removed. After each trial, the mouse was placed in a cage lined with absorbent paper towels and allowed to rest. *n* = 15 PBS-treated and *n* = 17 WNT3A-treated mice were used for WNT3A induction experiments in WT mice. *n* = 7 KO + PBS mice, *n* = 9 KO + IWP2 mice and *n* = 12 WT + PBS mice were used for rescue experiments in *Kdm5c* KO mice and WT controls as indicated.

Detailed two-way ANOVA analysis related to Fig. 5d: two-way ANOVA for hidden platform (H1–H5): *F*_(1.150)_ = 6,4707, *P* = 0.01198; and reversal (R1–R3): *F*_(1,96)_ = 6.5465, *P* = 0.01207.

Detailed two-way ANOVA analysis related to Fig. 5j: hidden platform (H1–H5) (two-way ANOVA: KO + PBS vs KO + IWP2, *F*_(1.70)_ = 7.6812, *P* = 0.007142; KO + IWP2 vs WT + PBS, *F*_(1.95)_ = 4.4292, *P* = 0.03797; KO + PBS vs WT + PBS, *F*_(1.85)_ = 29.2376, *P* = 0.0000005779). Visible platform (V1–V2) (two-way ANOVA: KO + PBS vs KO + IWP2, *F*_(1.28)_ = 0.1908, *P* = 0.6656; KO + IWP2 vs WT + PBS, *F*_(1.38)_ = 20.1275, *P* = 0.00006509; KO + PBS vs WT + PBS *F*_(1.34)_ = 28.7147, *P* = 0.000005877). Reversal platform (R1–R3) almost reached significance in the KO + PBS vs KO + IWP2 comparison (two-way ANOVA: KO + PBS vs KO + IWP2, *F*_(1.42)_ = 2.9032, *P* = 0.09579; KO + IWP2 vs WT + PBS, *F*_(1.57)_ = 2.2712, *P* = 0.1373; KO + PBS vs WT + PBS, *F*_(1.51)_ = 10.6433, *P* = 0.001974).

### Spine density analysis

Spine density analysis of pyramidal cells in the CA1, the PFC and the BLA was performed in mice after in utero intracerebroventricular injections of PBS or WNT3A in E13.5 WT embryos. Brains from adult (aged 4–7 months) mice were dissected and incubated in Goldi-Cox solution according to the protocol supplied by Neurodigitech. After 2 weeks, brains were sent to Neurodigitech and investigated. In brief, each brain sample was composed of 6–8 slides that covered the range of the regions of interest (ROIs), colour-coded with the in-house alphanumerical coding system, randomly assigned to each slide folder and then distributed to the analysts who were blinded to the original slide identities. The slides included serial coronal sections that covered the anterior-to-posterior axis of the brain. The sampling of ROIs included basal and apical dendrites of pyramidal cells in the BLA, the PFC (layer III/IV) and the CA1 of the hippocampus. The dendritic segments of ROIs were then chosen and analysed using a stereology-based software called Neurolucida (MBF Bioscience), installed on a Dell PC workstation that included a Nikon Eclipse Ni microscope with a Hamamatsu CCD camera (C11440, ORCA-Flash4.0) (full resolution of 2,048 × 2048 pixels), motorized *x, y* and *z* focus for high-resolution image acquisition and digital quantitation. The following criteria were applied for selecting candidate neurons for analysis: (1) visualization of fully filled soma with no overlap of neighbouring soma and fully filled dendrites; (2) tapering of the most distal dendrites; and (3) visualization of the complete 3D profile of dendritic tress using the 3D display of the imaging software. After tracing and spine counting, the raw data were extrapolated and quantified using the NeuroExplorer program (MBF Bioscience) followed by statistical analysis (one-way ANOVA followed by Tukey’s multiple comparison test: *P* < 0.05 was considered significant. *n* = 7 PBS-treated mice for basal spine density analysis, *n* = 6 PBS-treated mice for apical spine density analysis, *n* = 4 mice for lower WNT inhibitor concentration (33 ng) and *n* = 3 mice for higher WNT inhibitor concentration (50 ng).

## Extended Data

## Supplementary Material

Supplementary Table 1 and 2

Supplementary Figure 1

## Data Availability

Data have been deposited into public databases. All software and codes are publicly available. RNA-seq data (Fig. 2 and Extended Data Fig. 5) were mapped against the human genome version hg19. CUT&RUN reads were mapped to the human reference genome hg38. RNA-seq data (Fig. 4 and Extended Data Fig. 7) were aligned to the human reference genome hg38. ATAC–seq reads were mapped to the human genome hg38. The RNA-seq data from Fig. 2 and Extended Data Fig. 5 are accessible at the ArrayExpress Archive under accession number E-MTAB-7551. The following data are accessible from the Gene Expression Omnibus database: CUT&RUN (accession number GSE210857); RNA-seq data from Fig. 4 and Extended Data Fig. 7 (accession number GSE211063); ATAC–seq data (accession number GSE210090). GSE210857, GSE211063 and GSE210090 are combined into a SuperSeries and are accessible under GSE239733. Source data are provided with this paper.
