# Supplementary material for: WNT signalling control by KDM5C during development affects cognition
Source: Nature. Author manuscript; Available in PMC 2024 Apr 24. (PMC10954547; doi:10.1038/s41586-024-07067-y)
Supplement: Supplementary Table 1 and 2 [file NIHMS1972710-supplement-Supplementary_Table_1_and_2.pdf]

Antibody name	Company	Catalog number	Dilution
Oct3/4 (C-10)	Santa Cruz Biotechnology	sc-5279	1:500
Sox-2 (E-4)	Santa Cruz Biotechnology	sc-365823	1:400
TRA-1-60	eBioscience	13-8863-82	1:500
Pax6	Biolegend	901301	1:80
Nestin	BD Biosciences	611659	1:100
ZO1-1A12	Invitrogen	33-9100	1:200
Ki-67	DAKO	M-7240	1:500
Tbr1	EMD Millipore	AB10554	1:400
Tbr2	Abcam	ab23345	1:100
Tubulin β3	Covance	MMS-435P	1:500
Ctip2	Abcam	ab18465	1:250
Satb2	Abcam	ab92446	1:80
Histone H3 (D1H2)	CST	4499	1:1000
GAPDH (14C10)	CST	2118	1:1000
β-Catenin	BD Biosciences	610153	1:1000
KDM5C (house made)	Yang Shi laboratory<br>(Boston Children’s Hospital, HMS)	N/A	1/1000
JARID1C	Bethyl Laboratories	A301-034A	1 μg per reaction
Goat anti-rabbit IgG (H+L) 488	Thermo Fisher Scientific	A11034	1:1000

Goat anti-rabbit IgG (H+L) 594	Thermo Fisher Scientific	A11012	1:1000
Goat anti-mouse IgG (H+L) 488	Thermo Fisher Scientific	A11001	1:1000
Goat anti-mouse IgG (H+L) 555	Thermo Fisher Scientific	A32727	1:1000
Goat anti-rat IgG (H+L) 488	Thermo Fisher Scientific	A11006	1:1000
Goat anti-rabbit IgG Antibody (H+L) HRP	Millipore	AP307P	1:1000
Goat anti-mouse IgG Antibody (H+L) HRP	Millipore	AP308P	1:1000
Anti-rabbit	EpiCypher	13-0047	0.5 µg per reaction.

Table 1. List of antibodies used in this study.

Target	Forward sequence	Reverse sequence
GAPDH	TGGTCTCCTCTGACTTCAACAGCG	AGGGGTCTACATGGCAACTGTGAG
GAPDH	CACCACACTGAATCTCCCCT	TGGTTGAGCACAGGGTACTT
Oct4	GTACTCCTCGGTCCCTTTCC	CAAAAACCCTGGCACAAACT
Nanog	TGAACCTCAGCTACAAACAG	TGGTGGTAGGAAGAGTAAAG
Sox2	AACCCCAAGATGCACAACT	GCTTAGCCTCGTCGATGAAC
Pax6	GTGTCCAACGGATGTGTGAG	CTAGCCAGGTTGCGAAGAAC
Nestin	CAGCGTTGGAACAGAGGTT	TGGCACAGGTGTCTCAAGG
Tbr2	CGCCACCAAACTGAGATGAT	CACATTGTAGTGGGCAGTGG
TUJI	GTATCCCGACCGCATCAT	TCTCATCCGTGTTCTCCA
Ctip2	CGGGCGATGCCAGAATAGAT	TGATGAGCTCCCTCTGGGAC
Satb2	AGTTGGACGGCTCTCTTGAA	CCACCTTCCCAGCTTGATTA
Wnt1	CGCTGGAACTGTCCCAC	AACGCCGTTTCTCGACAG
Wnt3a	TCCCACACCGTCAGGTACTC	CAGCTGAGCAGAGCAGAGAC
Axin2	TGGGATGATCTGTTGCAGAG	CAGTGATTCTAGCAGGCCTCA

Table 2. List of oligonucleotides used in this study.
