# Supplementary material for: WNT signalling control by KDM5C during development affects cognition
Source: Nature. Author manuscript; Available in PMC 2024 Apr 24. (PMC10954547; doi:10.1038/s41586-024-07067-y)
Supplement: Supplementary Figure 1 [file NIHMS1972710-supplement-Supplementary_Figure_1.pdf]

nature portfolio

https://doi.org/10.1038/s41586-024-07067-y

Supplementary information

WNT signalling control by KDM5C during development affects cognition

In the format provided by the authors and unedited

Extended Data Figure 1b

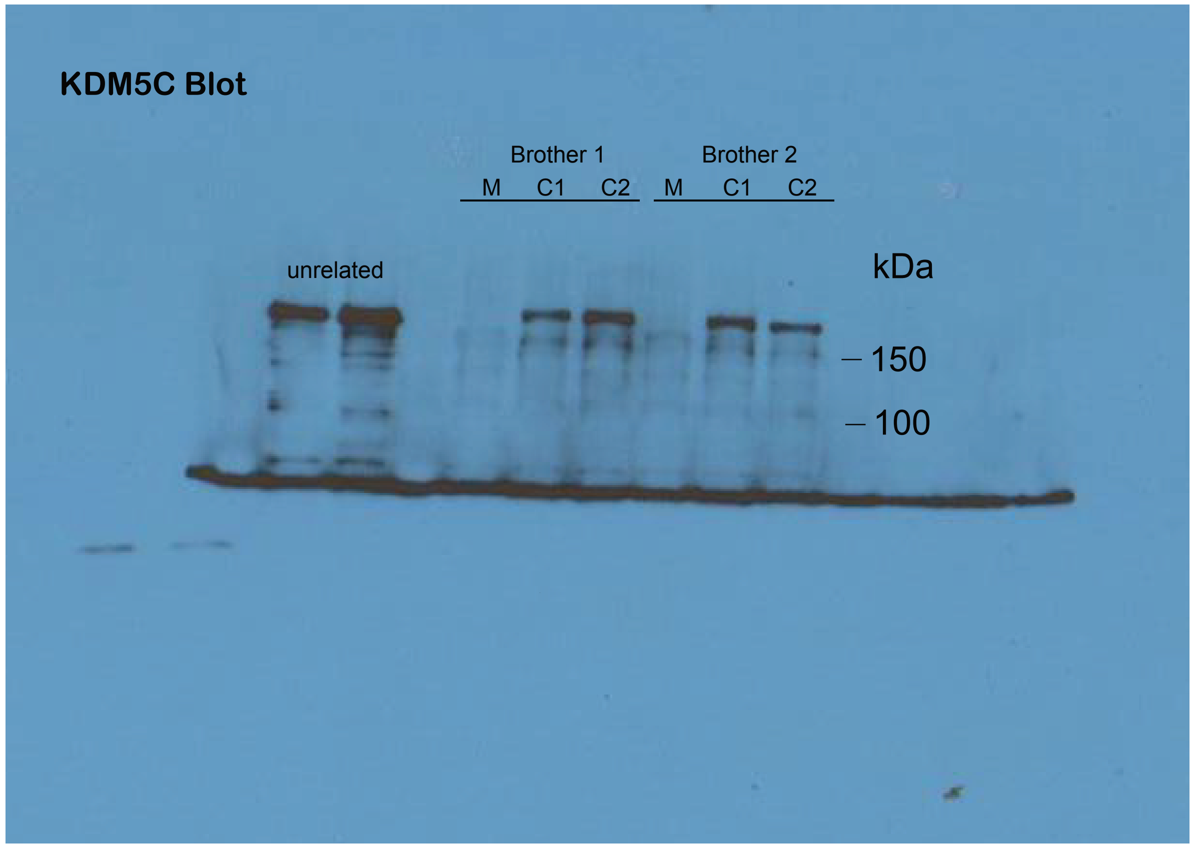

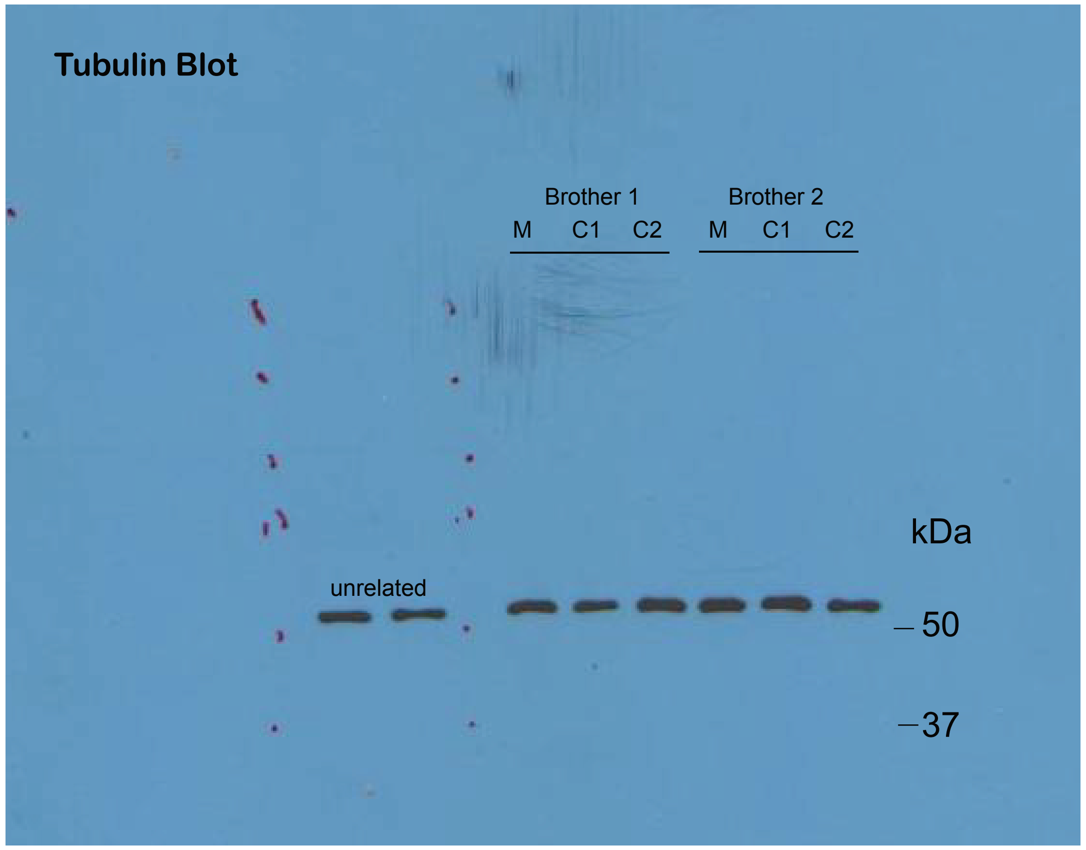

The same amounts of quantitated cell lysates were loaded onto two separate electrophoretic gels—one for KDM5C and another for Tubulin. Importantly, these gels were processed identically and were then blotted and probed with KDM5C and Tubulin antibodies, respectively. In general, it's best to have the blot from a single gel to be probed with two different antibodies, which often involves cutting the blot into two pieces, one probed with the antibody of interest and the other, the loading control antibody. However, in this case, we don't want to cut the blot because we wanted to see the entire lane stained with the KDM5C antibody in order to determine if there are any non-specific cross-reactivities of the KDM5C antibodies by comparing the Corrected cells with Mutant cells. It should be noted that since we loaded the same amount of lysates, ran the two gels and blotted them at the same time, it minimized the risk of artifacts. We therefore believe the Western blots have faithfully reflected the KDM5C expression levels in wildtype versus KDM5C KO cells.

Extended Data Figure 4m, part 1

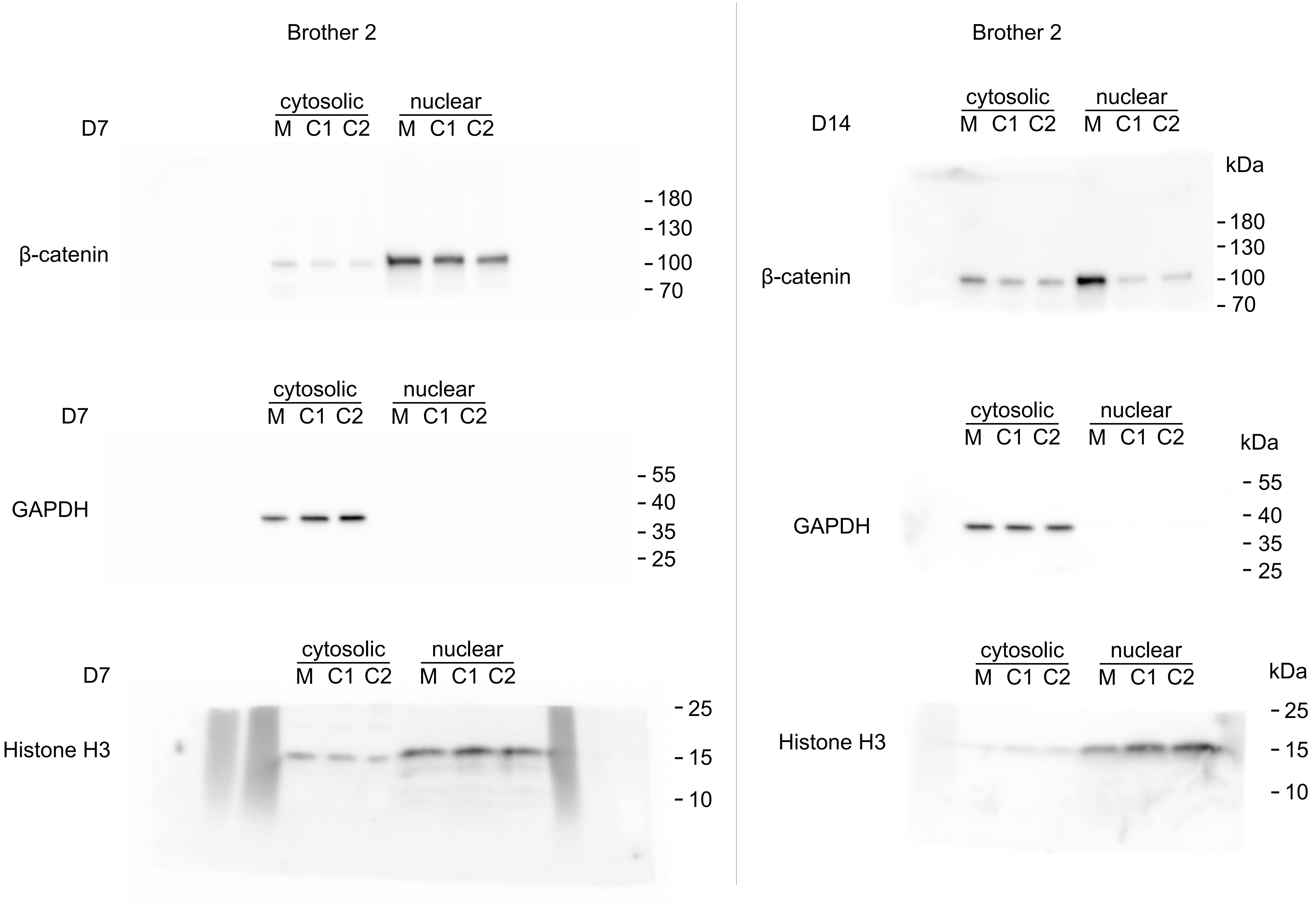

β-Catenin, GAPDH and Histone H3 were run on the same gel. Blots were cut at the indicated markers. First cut was performed between 70-55 kDa and the second around 25 kDa.

Extended Data Figure 4m, part 2

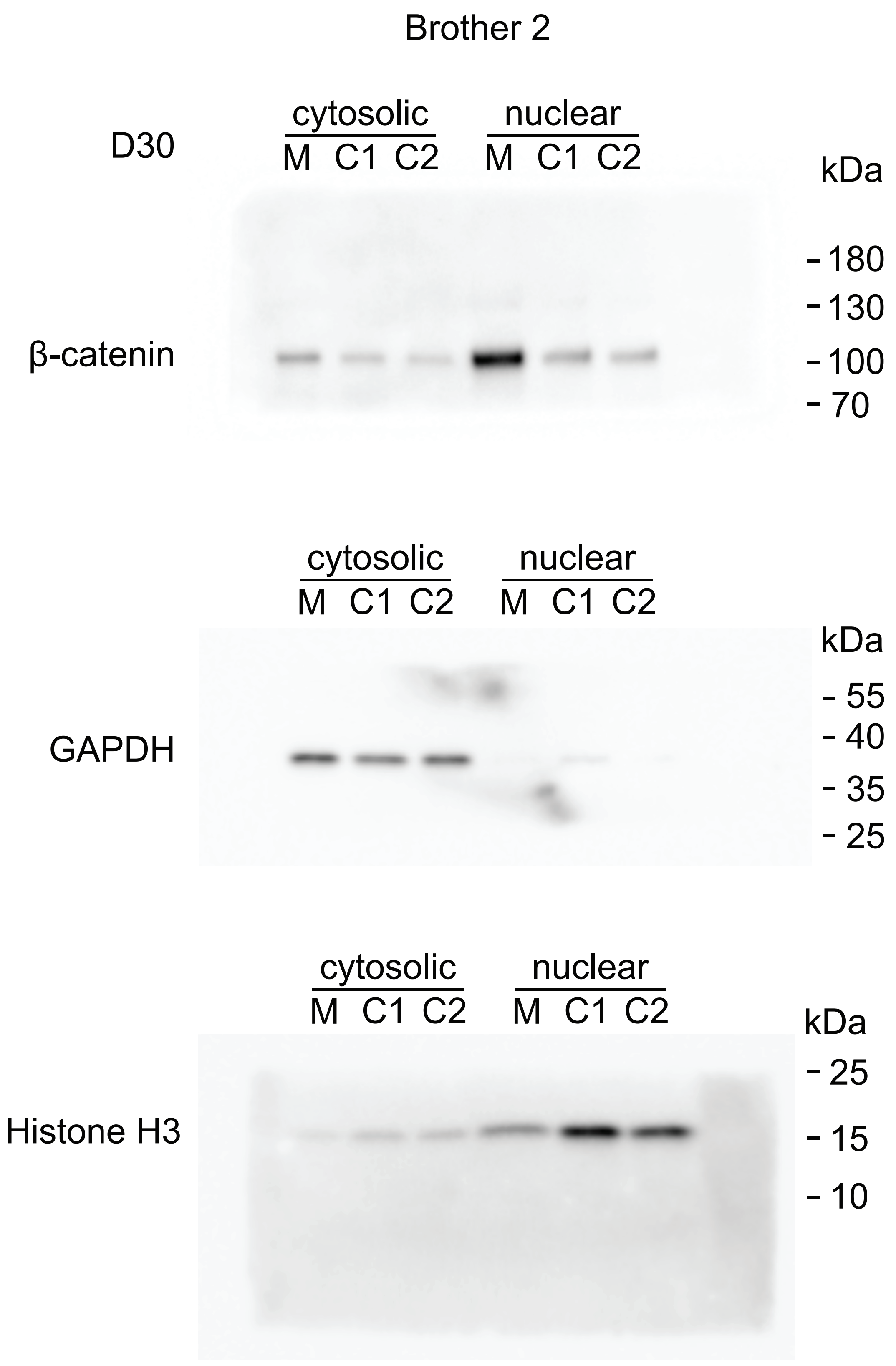

β-Catenin, GAPDH and Histone H3 were run on the same gel. Blots were cut at the indicated markers. First cut was performed between 70-55 kDa and the second around 25 kDa.

Extended Data Figure 6c, part 1

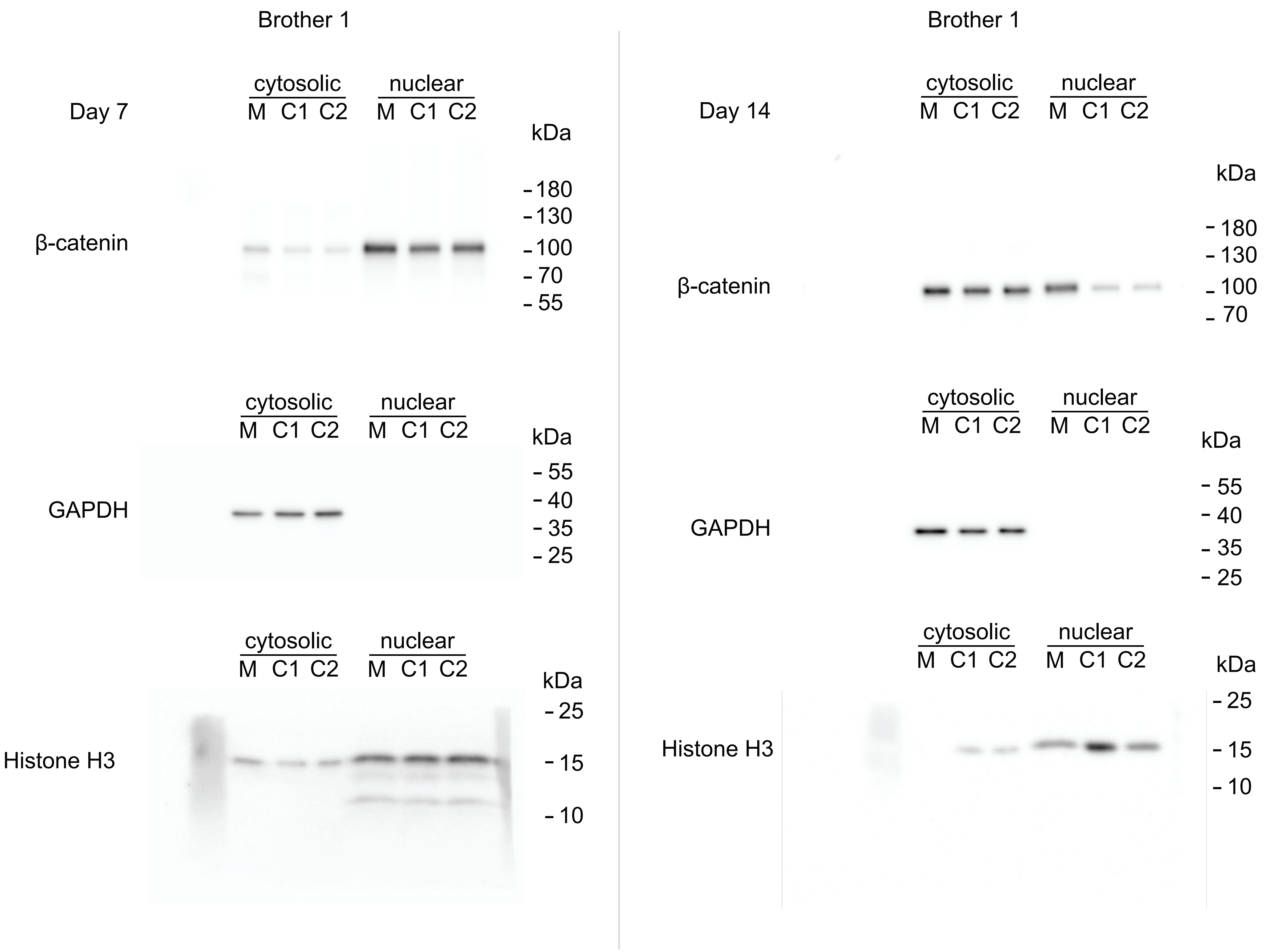

β-Catenin, GAPDH and Histone H3 were run on the same gel. Blots were cut at the indicated markers. First cut was performed between 70-55kDa and the second around 25 kDa.

Extended Data Figure 6c, part 2

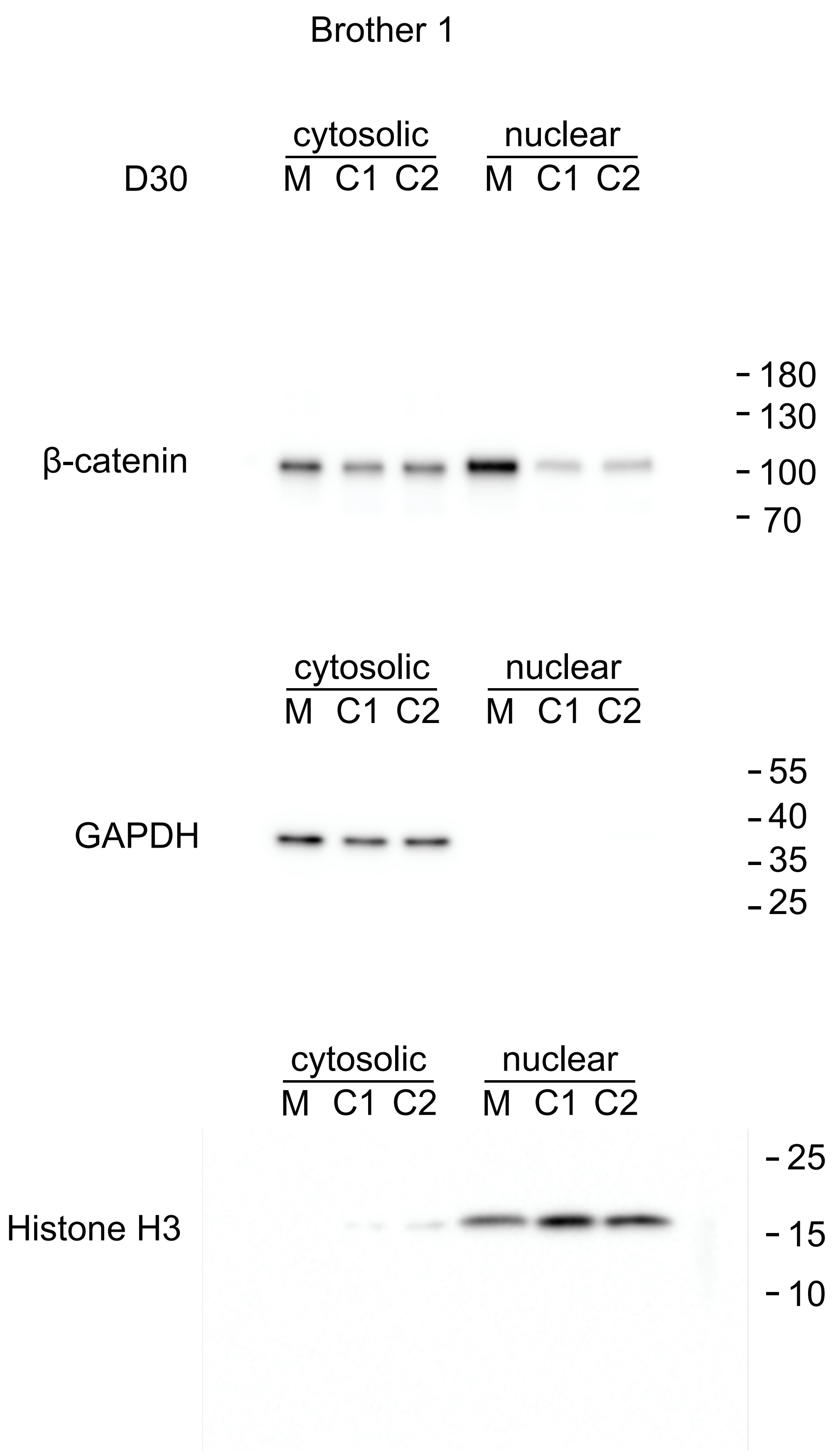

β-Catenin, GAPDH and Histone H3 were run on the same gel. Blots were cut at the indicated markers. First cut was performed between 70-55 kDa and the second around 25 kDa.
