# Supplementary material for: WNT signalling control by KDM5C during development affects cognition
Source: Nature. 2024 Feb 21;627(8004):594–603. doi: 10.1038/s41586-024-07067-y (PMC10954547; doi:10.1038/s41586-024-07067-y)
Supplement: Supplementary file 1 [file 41586_2024_7067_MOESM2_ESM.pdf]

nature portfolio

Corresponding author(s): Yang Shi and Violetta Karwacki-Neisius

Last updated by author(s): 11.08.2023

Reporting Summary

Nature Portfolio wishes to improve the reproducibility of the work that we publish. This form provides structure for consistency and transparency in reporting. For further information on Nature Portfolio policies, see our Editorial Policies and the Editorial Policy Checklist.

Statistics

For all statistical analyses, confirm that the following items are present in the figure legend, table legend, main text, or Methods section.

n/a	Confirmed	
☐	☒	The exact sample size (n) for each experimental group/condition, given as a discrete number and unit of measurement
☐	☒	A statement on whether measurements were taken from distinct samples or whether the same sample was measured repeatedly
☐	☒	The statistical test(s) used AND whether they are one- or two-sided<br>*Only common tests should be described solely by name; describe more complex techniques in the Methods section.*
☐	☒	A description of all covariates tested
☐	☒	A description of any assumptions or corrections, such as tests of normality and adjustment for multiple comparisons
☐	☒	A full description of the statistical parameters including central tendency (e.g. means) or other basic estimates (e.g. regression coefficient) AND variation (e.g. standard deviation) or associated estimates of uncertainty (e.g. confidence intervals)
☐	☒	For null hypothesis testing, the test statistic (e.g. F, t, r) with confidence intervals, effect sizes, degrees of freedom and P value noted<br>*Give P values as exact values whenever suitable.*
☒	☐	For Bayesian analysis, information on the choice of priors and Markov chain Monte Carlo settings
☒	☐	For hierarchical and complex designs, identification of the appropriate level for tests and full reporting of outcomes
☒	☐	Estimates of effect sizes (e.g. Cohen's d, Pearson's r), indicating how they were calculated

Our web collection on statistics for biologists contains articles on many of the points above.

Software and code

Policy information about availability of computer code

Data collection	Libraries for RNA-seq, CUT&RUN and ATAC-seq were sequenced on HiSeq 2500 (illumina), NextSeq500 (Illumina) and Next Seq 550 (Illumina). Q-PCR data were collected on a Roche LightCycler 480 Instrument. RNA-Seq data were mapped against the human genome version hg19 with STAR-2.5.2b. CUT&RUN reads were mapped to hg38 using Bowtie2.3.5.1. Demultiplexed data for RNA-seq were align to human reference genome hg38 using HISAT (v2.1.0). ATAC-seq reads were mapped to the human genome (build GRCh38/hg38) using Bowtie2. Immunoblot image was required with ChemiDoc™ MP Imaging System (Bio-rad, 12003154). Luminescence in Luciferase assays was measured using the EnSight Multimode Plate Reader (PerkinElmer). Images were captured suing the Nikon Eclipse TE2000-U inverted Fluorescence microscope. Nikon Eclipse Ni microscope with Hamamatsu CCD camera (C11440, ORCA-Flash4.0) was used for spine density analysis.
Data analysis	The following custom and publicly-available software packages were used and are detailed in the Methods section: Bowtie2.3.5.1, STAR-2.5.2b, R-3.4.1, Bioconductor 3.6, R package Genomic Alignments 1.14.0, DESeq2 1.18.0, ggplot2_2.2.1, goseq 1.28.0 package, BamCovergae in Deeptools3.3.1, MACS2.2.6, Bedtools 2.28.0, GraphPad Prism 10.0.3, Homer-4.11, Prism9, HISAT 2.1.0, Cufflinks 2.1.0, Qlucore Omics Exploere 3.8, MACS 2.1.0, FASTQC 0.11.8, bcbio-nextgen 1.2.8, Atropos 1.1.29, Bowtie2 2.4.1, Bowtie2.3.5.1, Sambamba 0.7.1, Samtools 1.9, MACS2 2.2.7.1, ataqv 1.2.1, IGV 2.11.9, IGV 2.8.4, BEDTools 2.27.1, R 3.6.1, DESeq2 1.30.1, ChIPseeker 1.26.2 and ClusterProfiler 3.18.1. Noldus Etho-Vision XT video tracking software 15.0, Noldus Etho-Vision XT video tracking software 12.0, Neurolucida (MBF Bioscience, VT), NeuroExplorer program (MBF Bioscience, VT).

For manuscripts utilizing custom algorithms or software that are central to the research but not yet described in published literature, software must be made available to editors and reviewers. We strongly encourage code deposition in a community repository (e.g. GitHub). See the Nature Portfolio guidelines for submitting code & software for further information.

Data

Policy information about availability of data

All manuscripts must include a data availability statement. This statement should provide the following information, where applicable:

- Accession codes, unique identifiers, or web links for publicly available datasets
- A description of any restrictions on data availability
- For clinical datasets or third party data, please ensure that the statement adheres to our policy

Data have been deposited in public data bases. All software and codes are publicly available. RNA-Seq data (Figure 2 and Extended Data Fig. 5) were mapped against the human genome version hg19. CUT&RUN reads were mapped to the human reference genome hg38. RNA-seq data (Figure 4 and Extended Data Fig. 7) were align to the human reference genome hg38. ATAC-seq reads were mapped to the human genome hg38. The RNA-seq data from Figure 2 and Extended Data Fig. 5 are accessible at the ArrayExpress Archive under accession number E-MTAB-7551 (https://www.ebi.ac.uk/biostudies/arrayexpress/studies/E-MTAB-7551). CUT&RUN data is accessible under the accession number GSE210857 (https://www.ncbi.nlm.nih.gov/geo/query/acc.cgi?acc=GSE210857). RNA-seq data from Figure 4 and Extended Data Fig. 7 are accessible under the accession number GSE211063 (https://www.ncbi.nlm.nih.gov/geo/query/acc.cgi?acc=GSE211063). ATAC-seq data are accessible under the accession number GSE210090 (https://www.ncbi.nlm.nih.gov/geo/query/acc.cgi?acc=GSE210090). GSE210857, GSE211063 and GSE210090 are combined into a SuperSeries and are accessible under GSE239733 (https://www.ncbi.nlm.nih.gov/geo/query/acc.cgi?acc=GSE239733). Source data have been provided as Source Data files in the manuscript.

Human research participants

Policy information about studies involving human research participants and Sex and Gender in Research.

Reporting on sex and gender	N/A
Population characteristics	N/A
Recruitment	N/A
Ethics oversight	N/A

Note that full information on the approval of the study protocol must also be provided in the manuscript.

Field-specific reporting

Please select the one below that is the best fit for your research. If you are not sure, read the appropriate sections before making your selection.

☒ Life sciences ☐ Behavioural & social sciences ☐ Ecological, evolutionary & environmental sciences

For a reference copy of the document with all sections, see nature.com/documents/nr-reporting-summary-flat.pdf

Life sciences study design

All studies must disclose on these points even when the disclosure is negative.

Sample size	No sample size calculation was performed. The number of samples in each experiment was determined based on standard practice in the field. The majority of our data is performed as N = or > 3 independent experiments unless otherwise stated as for example for some sequencing data (ATAC-seq and CUT&RUN data) and Immunoblots that require less replicates (N=2). For in vivo studies a minimum of 3 replicates were performed. (Shen et al. 2016. Cell; Paulsen et al. 2022. Nature; Brookes et al. 2015. Human Molecular Genetics; Iwase et al., 2016. Cell Reports). The number of independent experiments are indicated in the legend of each Figure and the Methods section.
Data exclusions	Mice that were sick or developed surgery related injuries were euthanized and excluded to ensure that only healthy mice were part of our study.
Replication	All attempts of replication were successful and described in the legends and Methods section.
Randomization	For all studies, samples (cells, tissues, mice) were allocated randomly to experimentation type (Immunostaining, Sequencing data etc.) and were only segregated by genotype or treatment. Both males and females were included in behavioral studies for Wnt3a induction..
Blinding	Investigators that performed behavioral studies were all blinded to the study groups being analyzed. All other analysis including bioinformatic analysis were applied the same to all samples without adjustment for genotype or treatment.

Reporting for specific materials, systems and methods

We require information from authors about some types of materials, experimental systems and methods used in many studies. Here, indicate whether each material, system or method listed is relevant to your study. If you are not sure if a list item applies to your research, read the appropriate section before selecting a response.

Materials & experimental systems

n/a	Involved in the study	
☐	☒	Antibodies
☐	☒	Eukaryotic cell lines
☒	☐	Palaeontology and archaeology
☐	☒	Animals and other organisms
☒	☐	Clinical data
☒	☐	Dual use research of concern

Methods

n/a	Involved in the study	
☒	☐	ChIP-seq
☒	☐	Flow cytometry
☒	☐	MRI-based neuroimaging

Antibodies

Antibodies used

Primary antibodies:
Oct3/4 Antibody (C-10) (Santa Cruz Biotechnology sc-5279); 1:500
Sox-2 Antibody (E-4) (Santa Cruz Biotechnology sc-365823) 1: 400
TRA-1-60 (Podocalyxin) Monoclonal Antibody (eBioscience13-8863-82) 1:500
Purified anti-Pax6 Antibody (Biolegend 901301); 1:80
Purified mouse anti-Nestin (BD Biosciences 611659) 1:100
ZO-1 Monoclonal Antibody (ZO1-1A12) (Invitrogen 33-9100) 1:200
Ki-67 Antigen, MIB-1, Unconjugated (DAKO M-7240) 1:500
Anti-Tbr1 Antibody (EMD Millipore AB10554); 1.400
Anti-Tbr2/Eomes antibody (Abcam ab23345); 1:100
Purified anti-Tubulin β3 (TUBB3) Antibody (Covance MMS-435P); 1:500
Anti-Ctip2 antibody (25B6) (Abcam ab18465); 1:250
Recombinant Anti-SATB2 antibody (EPNCIR130A) (Abcam ab92446); 1:80
For Western Blot: Anti-KDM5C house made; 1:1000
Histone H3 (D1H2), (CST 4499) 1:1000
GAPDH (14C10), (CST 2118) 1:1000
β-Catenin (BD Biosciences 610153) 1:1000
For CUT&RUN: Rabbit anti-JARID1C Antibody, Affinity Purified (Bethyl Laboratories A301-034A); 1 µg per reaction.

Secondary antibodies:
Goat anti-Rabbit IgG (H+L) Seconday antibody, Alexa Fluor 488 (Thermo Fisher Scientific A11034); 1:1000
Goat anti-Rabbit IgG (H+L) Seconday antibody, Alexa Fluor 594 (Thermo Fisher Scientific A11012); 1:1000
Goat anti-mouse IgG (H+L) Seconday antibody, Alexa Fluor 488 (Thermo Fisher Scientific A11001); 1:1000
Goat anti-mouse IgG (H+L) Seconday antibody, Alexa Fluor 555 (Thermo Fisher Scientific A32727); 1:1000
Goat anti-rat IgG (H+L) Seconday antibody, Alexa Fluor 488 (Thermo Fisher Scientific A11006); 1:1000
For Western Blot: Goat anti-Rabbit IgG Antibody (H+L) HRP conjugate (Millipore AP307P); 1:1000
Goat anti-Mouse IgG Antibody (H+L) HRP conjugate (Millipore AP308P); 1:1000
For CUT&RUN: Anti-Rabbit Secondary antibody for CUTANA CUT&Tag Workflows (EpiCypher 13-0047); 0.5 µg per reaction.

Validation

All the primary antibodies were validated by the manufacturers. Despite the fact that each of the antibodies are well cited we test antibodies in tissues that do not show their expression or upon knockdown or knockout of protein if possible. In addition we establish gradient concentration testing and control for expression pattern and the background of the secondary antibodies. The validations and citations of each antibody according to the manufacturers website are listed below:
The Oct3/4 Antibody (C-10) (Santa Cruz Biotechnology sc-5279) previously validated in human for IF as stated by the manufactures on their website (https://www.scbt.com/p/oct-3-4-antibody-c-10). Also validated in Sharma, SD. et al. 2023. Cell Rep. Overall cited in 2.512 publications.
The Sox-2 Antibody (E-4) (Santa Cruz Biotechnology sc-365823) previously validated in human for IF as stated by the manufactures on their website (https://www.scbt.com/p/sox-2-antibody-e-4). Also validated in Mun, D. et al. 2022. Stem Cell. Overall cited in 290 publications.
TRA-1-60 (Podocalyxin) Monoclonal Antibody (eBioscience13-8863-82) previously validated in human for IF as stated by the manufactures on their website (https://www.thermofisher.com/antibody/product/TRA-1-60-Podocalyxin-Antibody-clone-TRA-1-60-Monoclonal/13-8863-82). Also validate in Tsanov et al., 2017. Nature Cell Biology. Overall cited in 18 publications.
Purified anti-Pax6 Antibody (Biolegend 901301) previously validated in human for IF as stated by the manufactures on their website (https://www.biolegend.com/en-us/cell-health/purified-anti-pax-6-antibody-11511?GroupID=GROUP26). Also Ziffra, et al. 2021. Nature. Overall cited in 308 publications.
Purified mouse anti-Nestin ((10c2): sc-23927) previously validated in human for IF as stated by the manufactures on their website (https://www.scbt.com/p/nestin-antibody-10c2?gclid=EAIaIQobChMI4dio-sOrggMVCElHAR1czgegEAAYAiAAEgKJU_D_BwE#citations). Also validated in Vinci, L. et al. 2016. Eur J Histochem. Overall cited in 228 publications.
ZO-1 Monoclonal Antibody (ZO1-1A12) Invitrogen 33-9100 previously validated in human for IF as stated by the manufactures on their website (https://www.thermofisher.com/antibody/product/ZO-1-Antibody-clone-ZO1-1A12-Monoclonal/33-9100?ef_id=EAIaIQobChMIwe7Z7cmrggMViOnICh1oNgFEEAAYAiAAEgJWYvD_BwE:G:s&s_kwcid=AL!3652!3!459737518508!!!g!!!109508257751065313204068&cid=bid_pca_aup_r01_co_cp1359_pjt0000_bid00000_0se_gaw_dy_pur_con&gclid=EAIaIQobChMIwe7Z7cmrggMViOnICh1oNgFEEAAYAiAAEgJWYvD_BwE). Also validated in Vatine et al., 2019, Cell Stem Cell. Overall cited in 603 publications.

Ki-67 Antigen, MIB-1, Unconjugated (DAKO M-7240) previously validated in human for IHC as stated by the manufactures on their website (https://www.agilent.com/en/product/immunohistochemistry/antibodies-controls/primary-antibodies/ki-67-antigen-%28concentrate%29-76646). Also validate in Gerdes et al., 1992, J Pathology. Overall cited in 4000 publications.
Anti-Tbr1 Antibody (EMD Millipore AB10554) previously validated in mouse for IH(P) as stated by the manufactures on their website (https://www.emdmillipore.com/US/en/product/Anti-Tbr1-Antibody,MM_NF-AB10554). Also validated in Fernandes et al. 2012. Developmental biology. Overall cited in 36 publications.
Anti-Tbr2/Eomes antibody (Abcam ab23345) previously validated in mouse and human for IHC as stated by the manufactures on their website (https://www.abcam.com/products/primary-antibodies/tbr2--eomes-antibody-ab23345.html). Also validated in Shi et al. Nature protocols, 2012. Overall cited in 474 publications.
Purified anti-Tubulin β3 (TUBB3) Antibody (Covance MMS-435P) previously validated in mouse and human for IHC-P as stated by the manufactures on their website (https://www.biolegend.com/ja-jp/products/purified-anti-tubulin-beta-3-tubb3-antibody-11580?GroupID=GROUP686). Validated also in Hu X., et al. 2006. Nature Neuroscience and Shi et al. Nature protocols. 2012. Overall cited in 751 publications.
Anti-Ctip2 antibody (25B6) (Abcam ab18465) previously validated in mouse and human for IHC/IF as stated by the manufactures on their website (https://www.abcam.com/products/primary-antibodies/ctip2-antibody-25b6-ab18465.html). Validated also in Bandler et al. Nature. 2022 and Shi et al., Nature protocols, 2012. Overall cited in 718 publications.
Recombinant Anti-SATB2 antibody (EPNCIR130A) (Abcam ab92446) previously validated in mouse and human for IHC/IF as stated by the manufactures on their website (https://www.abcam.com/products/primary-antibodies/satb2-antibody-epncir130a-ab92446.html). Also validate in Wang et al. 2021. Development and Shi et al., Nature protocols, 2012. Overall cited in 55 publications.
Rabbit anti-JARID1C Antibody, Affinity Purified (Bethyl Laboratories A301-034A) previously validated in human for ChIP as stated by the manufactures on their website (https://www.thermofisher.com/antibody/product/JARID1C-Antibody-Polyclonal/A301-034A). Validated also in Outchkourov et al. Cell Reports 2013. Overall cited in 8 publications.
House made anti-KDM5C was raised by immunizing rabbits with the C-terminal segment of human KDM5C (1459–1559 amino acid, NP_001269551), which was expressed and purified as a histidine-tagged protein in E.coli. Resultant serum was affinity purified using the immunized protein as the ligand. Specificity was validated for WB using KDM5C—knockout mouse embryonic stem cells and neurons (Iwase et al., 2016. Cell Reports), shRNA-treated mouse embryonic fibroblasts, and human patient fibroblasts with nonsense mutations in KDM5C (Brookes et al., 2015. Hum Mol Genet). This antibody was made in the yang Shi laboratory, Boston Children's Hospital, Harvard Medical School.
Histone H3 (D1H2), (CST 4499) previously validated in human for WB as stated by the manufactures on their website (https://www.cellsignal.com/products/primary-antibodies/histone-h3-d1h2-xp-rabbit-mab/4499). Also validated in Al Shboul et al. 2023 Science reports. Overall cited in 1545 publications.
GAPDH (14C10), (CST 2118) previously validated in human for WB as stated by the manufactures on their website (https://www.cellsignal.com/products/primary-antibodies/gapdh-14c10-rabbit-mab/2118). Also validated in Vincent et al. 2023 BMC Cancer. Overall cited in 7616 publications.
β-Catenin (BD Biosciences 610153) previously validated in human for WB as stated by the manufactures on their website (https://www.bdbiosciences.com/en-us/products/reagents/microscopy-imaging-reagents/immunofluorescence-reagents/purified-mouse-anti-catenin.610153). Also validated in Eger et al. 2000. J Cell Biology. Overall cited in 6 publications.

Eukaryotic cell lines

Policy information about cell lines and Sex and Gender in Research

Cell line source(s)	We obtained human fibroblast cells that contain the c.2T>C mutation in KDM5C from our co-author Katrin Õunap and reprogrammed them to iPSC cells.
Authentication	The authentication of our lines was established by genotyping and Sanger sequencing. In addition Western Blot analysis was performed to confirm protein expression. Karyotyping was performed routinely to ensure a normal karyotype during experiments.
Mycoplasma contamination	All cell lines tested negative for mycoplasma. Mycoplasma testing was performed routinely during experiments to ensure a healthy state of cells.
Commonly misidentified lines (See ICLAC register)	No misidentified lines were used in the study.

Animals and other research organisms

Policy information about studies involving animals; ARRIVE guidelines recommended for reporting animal research, and Sex and Gender in Research

Laboratory animals	KDM5C-KO mice (C57Bl/6 background) are described in detail in Iwase et al. 2016. Immunohistochemistry was performed at E13.5 in KDM5C-KO and WT siblings. Time-pregnant CD-1 dams (E10) were obtained from Charles River Laboratories. Surgeries were performed at E13.5 and behavioral studies were performed with 4-7 months old adult mice. Mice were housed in individually ventilated cages suing a 12-hour light/dark cycle and had access to water and food ad libitum. Mice were housed in temperatures of 65-75F with 40-60% humidity.
Wild animals	This study did not involve wild animals.
Reporting on sex	For immunohistology E13.5 KDM5C-KO (males) and WT (males) siblings were used as female KDM5C-KO mice do not survive in utero. KDM5C-KO mice are described in detail in Iwase et al., 2016.<br>For Wnt3a induction experiments and the following behavioral experiments, both males and females adult CDl mice were used. For behavioral rescue experiments in KDMSC KO mice and WT controls only males were used as they show the strongest

behavioral impairments.

Field-collected samples	This study did not include samples collected from the field.
Ethics oversight	All animal studies were performed under the protocol approved by the Institutional Animal Care and Use Committee (IACUC) of the Boston Children's Hospital.

Note that full information on the approval of the study protocol must also be provided in the manuscript.
