# Supplementary material for: WNT signalling control by KDM5C during development affects cognition
Source: Nature. 2024 Feb 21;627(8004):594–603. doi: 10.1038/s41586-024-07067-y (PMC10954547; doi:10.1038/s41586-024-07067-y)
Supplement: Supplementary file 2 [file 41586_2024_7067_MOESM3_ESM.docx]

| Antibody name | Company | Catalog number | Dilution |
| --- | --- | --- | --- |
| Oct3/4 (C-10) | Santa Cruz Biotechnology | sc-5279 | 1:500 |
| Sox-2 (E-4) | Santa Cruz Biotechnology | sc-365823 | 1:400 |
| TRA-1-60 | eBioscience | 13-8863-82 | 1:500 |
| Pax6 | Biolegend | 901301 | 1:80 |
| Nestin | BD Biosciences | 611659 | 1:100 |
| ZO1-1A12 | Invitrogen | 33-9100 | 1:200 |
| Ki-67 | DAKO | M-7240 | 1:500 |
| Tbr1 | EMD Millipore | AB10554 | 1:400 |
| Tbr2 | Abcam | ab23345 | 1:100 |
| Tubulin β3 | Covance | MMS-435P | 1:500 |
| Ctip2 | Abcam | ab18465 | 1:250 |
| Satb2 | Abcam | ab92446 | 1:80 |
| Histone H3 (D1H2) | CST | 4499 | 1:1000 |
| GAPDH (14C10) | CST | 2118 | 1:1000 |
| β-Catenin | BD Biosciences | 610153 | 1:1000 |
| KDM5C (house made) | Yang Shi laboratory (Boston Children’s Hospital, HMS) | N/A | 1/1000 |
| JARID1C | Bethyl Laboratories | A301-034A | 1 μg per reaction |
| Goat anti-rabbit IgG (H+L) 488 | Thermo Fisher Scientific | A11034 | 1:1000 |
| Goat anti-rabbit IgG (H+L) 594 | Thermo Fisher Scientific | A11012 | 1:1000 |
| Goat anti-mouse IgG (H+L) 488 | Thermo Fisher Scientific | A11001 | 1:1000 |
| Goat anti-mouse IgG (H+L) 555 | Thermo Fisher Scientific | A32727 | 1:1000 |
| Goat anti-rat IgG (H+L) 488 | Thermo Fisher Scientific | A11006 | 1:1000 |
| Goat anti-rabbit IgG Antibody (H+L) HRP | Millipore | AP307P | 1:1000 |
| Goat anti-mouse IgG Antibody (H+L) HRP | Millipore | AP308P | 1:1000 |
| Anti-rabbit | EpiCypher | 13-0047 | 0.5 μg per reaction. |

**Table 1. List of antibodies used in this study.**

| Target | Forward sequence | Reverse sequence |
| --- | --- | --- |
| GAPDH | TGGTCTCCTCTGACTTCAACAGCG | AGGGGTCTACATGGCAACTGTGAG |
| GAPDH | CACCACACTGAATCTCCCCT | TGGTTGAGCACAGGGTACTT |
| Oct4 | GTACTCCTCGGTCCCTTTCC | CAAAAACCCTGGCACAAACT |
| Nanog | TGAACCTCAGCTACAAACAG | TGGTGGTAGGAAGAGTAAAG |
| Sox2 | AACCCCAAGATGCACAACT | GCTTAGCCTCGTCGATGAAC |
| Pax6 | GTGTCCAACGGATGTGTGAG | CTAGCCAGGTTGCGAAGAAC |
| Nestin | CAGCGTTGGAACAGAGGTT | TGGCACAGGTGTCTCAAGG |
| Tbr2 | CGCCACCAAACTGAGATGAT | CACATTGTAGTGGGCAGTGG |
| TUJI | GTATCCCGACCGCATCAT | TCTCATCCGTGTTCTCCA |
| Ctip2 | CGGGCGATGCCAGAATAGAT | TGATGAGCTCCCTCTGGGAC |
| Satb2 | AGTTGGACGGCTCTCTTGAA | CCACCTTCCCAGCTTGATTA |
| Wnt1 | CGCTGGAACTGTCCCAC | AACGCCGTTTCTCGACAG |
| Wnt3a | TCCCACACCGTCAGGTACTC | CAGCTGAGCAGAGCAGAGAC |
| Axin2 | TGGGATGATCTGTTGCAGAG | CAGTGATTCTAGCAGGCCTCA |

Table 2. List of oligonucleotides used in this study.
